# Randomised superiority trial evaluating an online transdiagnostic emotion regulation treatment for adolescents with mental health conditions: study protocol

**DOI:** 10.1136/bmjopen-2026-116511

**Published:** 2026-07-29

**Authors:** Katja Sjöblom, Martin Bellander, Jannike Nilbrink, Maria Zetterqvist, Filipa Sampaio, Erik Hedman-Lagerlöf, Clara Hellner, James J Gross, Hugo Hesser, Johan Bjureberg

**Affiliations:** 1 Centre for Psychiatry Research, Department of Clinical Neuroscience, Karolinska Institute, Stockholm, Sweden; 2 Stockholm Health Care Services, Region Stockholm, Stockholm, Sweden; 3 Clinical Department of Child and Adolescent Psychiatry in Linköping and Center for Social and Affective Neuroscience, Linkoping University Department of Biomedical and Clinical Sciences, Linköping, Sweden; 4 Department of Public Health and Caring Sciences, Uppsala University, Uppsala, Sweden; 5 Division of Psychology, Department of Clinical Neuroscience, Karolinska Institute, Stockholm, Sweden; 6 Department of Psychology, Stanford University, Stanford, California, USA; 7 School of Behavioural, Social and Legal Sciences, Örebro University, Örebro, Sweden

**Keywords:** Adolescent, Primary Care, Treatment Outcome, MENTAL HEALTH, HEALTH ECONOMICS, Clinical Trial

## Abstract

**Introduction:**

Mental health conditions are prevalent during adolescence, but access to early evidence-based treatments remains limited. Scalable, online, theory-based transdiagnostic interventions delivered in primary care have the potential to reduce this treatment gap, but the effectiveness remains unknown. The objective is to investigate whether an online transdiagnostic emotion regulation treatment for adolescents with mental health conditions is superior to an active control treatment.

**Methods and analysis:**

This single-blind, randomised clinical superiority trial will evaluate the effectiveness and cost-effectiveness of an online transdiagnostic emotion regulation treatment for adolescents with mental health conditions within primary care in Sweden. We aim to include 388 participants (adolescents aged 12–17 years with mental health conditions and their parents), recruited through primary care and self-referral. Patients will be randomised to either an online transdiagnostic emotion regulation treatment or an active control treatment. Participants will receive 6 weeks of therapist-guided online transdiagnostic emotion regulation treatment or an active control treatment consisting of 6 weeks of online supportive treatment. Parents will participate in parallel with their adolescents in both conditions and will receive an online parent course. The primary outcome, assessed by blinded assessors, will be clinical global symptom severity measured with the Clinical Global Impressions-Severity scale. Treatment effectiveness will be evaluated through blinded assessment and self-assessment at primary endpoint (immediately after treatment) and 3 months after treatment. In addition, a health economic evaluation will be conducted. The study will be undertaken between October 2023 and July 2026.

**Ethics and dissemination:**

The study has obtained ethical approval from the Swedish Ethical Review Authority. Findings will be disseminated in peer-reviewed publications and presented at scientific conferences.

**Trial registration number:**

NCT06067165.

**STRENGTHS AND LIMITATIONS OF THIS STUDY**

- Use of an active control condition, enhancing internal validity and accounting for nonspecific treatment effects.
- The use of assessors blinded to treatment allocation provides a more objective evaluation of treatment outcomes.
- Participants may be referred by a healthcare professional or self-referred, which may introduce selection bias and limit the representativeness of the sample.
- Due to the nature of psychological treatment, blinding of participants and therapists is not feasible, which may introduce bias.

## Introduction

 Increasing rates of mental health conditions and disorders among children and adolescents represent a growing global public health concern.^1 2^ Throughout the manuscript, we use the term ‘mental health conditions’ (trial registration and trial protocol: ‘mental health problems’). Despite the rising need, effective treatments for youth mental health conditions remain limited.^3^ Substantial barriers, including long waiting times and limited availability of professional services, prevent adolescents from accessing appropriate care.^4 5^

Primary care has the potential to deliver early interventions for adolescents with mental health conditions; when complemented by online treatment options, it can also help overcome key barriers to accessing psychological treatment.^6 7^ Online delivery provides a scalable and flexible format for implementing transdiagnostic interventions that target shared underlying mechanisms, such as emotion regulation, across mental health disorders.^8^ Difficulties in emotion regulation—characterised by impaired emotion regulation ability, frequent use of maladaptive strategies and infrequent use of adaptive ones—have been linked to the development and maintenance of a broad spectrum of mental health conditions.^9^ Although both prospective and experimental findings suggest that emotion regulation may play an important role in the emergence and maintenance of psychopathology, the nature of this relationship remains unclear. Intervention studies that experimentally manipulate emotion regulation processes, such as randomised controlled trials targeting emotion regulation, may be particularly valuable in clarifying this relationship.^9^

To meet this growing need, we developed a brief, online, transdiagnostic, therapist-guided emotion regulation treatment, the Primary Care Online Emotion-Regulation Treatment (POET). We recently found POET to be feasible and acceptable in a feasibility trial.^10^

In this protocol, we describe the design and methods of a parallel-group, single-blind randomised controlled trial (RCT) conducted within a primary care setting in Sweden, targeting adolescents with mental health conditions. The primary aim of the study is to evaluate whether POET is more effective than an active control treatment in clinical global symptom severity immediately after treatment. Secondary aims include examining durability of treatment effects on clinical global symptom severity and effects on emotion regulation and mental health symptoms and functioning immediately after treatment and 3 months after treatment, as well as conducting a health economic evaluation to determine whether POET is cost-effective relative to the active control treatment. We hypothesise that POET will demonstrate superior effects compared with the active control treatment immediately after treatment and at 3 months after treatment by (1) reducing clinical global symptom severity, (2) reducing maladaptive and enhancing adaptive emotion regulation, (3) improving mental health symptoms (depression, anxiety) and functioning and (4) demonstrating favourable cost-effectiveness.

Additional secondary aims, detailed in the trial protocol in the Supplement, include examining whether improvements in emotion regulation are a potential mechanism of change, identifying factors that influence for whom and under what conditions the treatment is most effective and further assessments of the durability of treatment effects and other distal outcomes.

## Methods and analysis

### Study design

**Figure 1:**
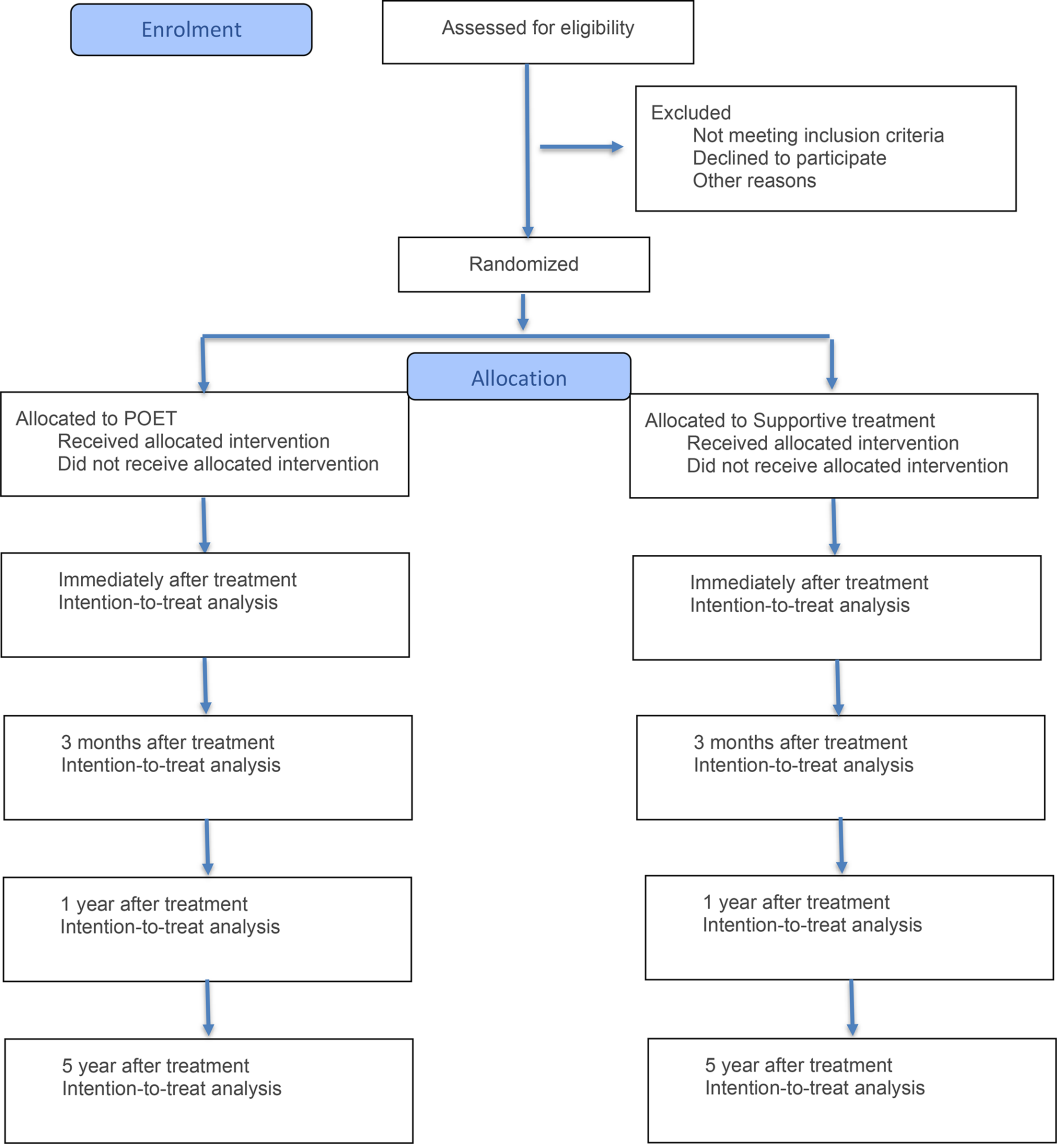
CONSORT 2025 flow diagram. CONSORT, Consolidated Standards of Reporting Trials; POET, Primary Care Online Emotion-Regulation Treatment.

The study is a 4-site parallel-group, single-blind RCT for adolescents with mental health conditions. Participants will be randomised to either receive POET or an active control treatment (hereafter, supportive treatment). Both groups will undergo 6 weeks of internet-delivered treatment with weekly modules and therapist support for youth and parents respectively. The primary endpoint is immediately after treatment. Participants are also followed up 3 months after treatment (secondary endpoint). The study began in October 2023 and is expected to end in July 2026. The current protocol follows the Standard Protocol Items: Recommendations for Interventional Trials (SPIRIT) guidelines for reporting a RCT.^11^ The full trial protocol is available in the Supplement. See figure 1 for a graphical summary of the trial design.

### Participants

We aim to recruit 388 Swedish adolescents with mental health conditions. Inclusion criteria are as follows: (1) age of 12–17 years (under 18), (2) presence of mental health conditions (defined as a Clinical Global Impressions–Severity (CGI-S) score of 2 or higher^12^ and (3) having at least one parent willing to participate in the parent course. Exclusion criteria include: (1) severe mental illness requiring specialised care or low global functioning corresponding to a Children’s Global Assessment Scale (CGAS)^13^ score of less than 41 on a scale of 1–100 (higher scores indicating better functioning), (2) acute suicidality (ongoing suicide plans or recent suicide attempt); (3) ongoing psychological treatment, (4) changes in psychopharmacological medication during the past 2 months, (5) insufficient Swedish comprehension and (6) life circumstances that could prevent treatment participation.

The selected age range of 12–17 years reflects both developmental and pragmatic considerations, excluding younger adolescents who would require more extensive parental involvement and older adolescents who would likely prefer to engage more independently in treatment.

### Recruitment and procedures

The study employs national recruitment of participants and involves clinicians working within primary care settings (ie, First-Line Mental Health Services) for adolescents across several regions in Sweden.^14^ Study coordination and supervision are based in Stockholm. The trial will be promoted within First-Line Mental Health Services and on social media. Individuals may be referred by a healthcare professional or refer themselves via the study website. Once referred, one parent will participate in an initial telephone screening to assess preliminary eligibility. If potentially eligible and interested, a therapist will conduct an assessment interview over video-link with the adolescent and at least one parent. The assessment is intended to evaluate all inclusion and exclusion criteria and consists of the semi-structured diagnostic interview The Mini-International Neuropsychiatric Interview for Children and Adolescents (MINI-KID)^15^ and a clinician-rated version of The Revised Child Anxiety and Depression Scale (RCADS).^16^ Prior to the inclusion assessment consent and baseline assessment are collected.

### Assessment points

Clinician assessments and self- and parent-reported questionnaires are administered before treatment (before randomisation), immediately after treatment and 3 months after treatment. Assessment points for secondary research questions are detailed in online supplemental file 1.

### Reimbursement

Adolescents will receive a gift card valued at 100 SEK (approximately £9) on completion of each assessment following the treatment period, with a maximum total reimbursement of 800 SEK (approximately £72). Participants will not receive any reimbursement during the treatment period.

### Randomisation and allocation concealment

Eligible participants will be randomly allocated (1:1) using block randomisation to either POET or Supportive treatment. An independent researcher will conduct the randomisation using an online randomisation system, which employs a secure pseudo-random number generator. Randomisation sequence will be concealed to assessors and study coordinators.

### Blinding

Primary and secondary clinician outcome measures will be assessed by an assessor blind to treatment allocation at baseline (before randomisation), immediately after treatment and 3 months after treatment. To ensure the integrity of blinding procedures, participants will be provided with explicit instructions not to reveal their treatment allocation to the blind assessors. After completing the assessment, the blind assessors guess the participant’s group allocation and disclose the reason for their guess (eg, true random guess) and report any disclosed group allocation. We will examine whether blinded assessors’ treatment allocation guesses differ from chance. Blind assessments will be audio-recorded to allow for calculation of inter-rater reliability (see online supplemental file 1). The participants, therapists and the study coordinators will not be blind to treatment allocation.

### Safety procedures

Self-harm will be monitored throughout the study using multiple methods, including clinician registration of negative events, such as self-harm during treatment and an adolescent self-reported adverse events measure administered after treatment. The Deliberate Self-Harm Inventory, Youth version,^17^ will be administered at follow-up assessments and blind assessors will alert the study coordinator if new onset or escalating self-harm is identified. Suicide risk will be monitored weekly through a self-assessed item monitoring suicidal ideation. During treatment, elevated scores on the suicidal ideation item will automatically trigger an alert, prompting the research team to, if necessary, initiate telephone contact with the adolescent or parent for a risk assessment. All therapists and blinded assessors will receive training in the assessment of suicide risk in adolescents and will adhere to standardised procedures if elevated suicide risk is identified during study period. Weekly team meetings will be held discussing safety issues and adverse events. Adverse events will be monitored and reported to the principal investigator throughout the treatment period. See Supplement for details.

### Planned interventions

Interventions in both conditions will be delivered across 6 weeks in a blended format combining six online text-based modules (available in audio format for adolescents) targeting both adolescents and their parents and one synchronous therapist-guided session delivered over video link. Both conditions include an asynchronous text-messaging function with the therapist, as well as regular support and feedback from a dedicated therapist. In addition, we have strived to incorporate a gender-neutral and non-heteronormative perspective in the development of the interventions. The interventions are detailed in the Supplement.

### POET

POET is theoretically grounded in the Extended Process Model of Emotion Regulation, which outlines the emotion-generative sequence and four families of regulation strategies: situational, attentional, cognitive and response modulation.^18 19^ Further, POET has been adapted from Internet-Delivered Emotion Regulation Individual Therapy for Adolescents for youth with nonsuicidal self-injury.^20^ POET was co-created with a patient involvement board (see Supplement). An initial version of the POET treatment was evaluated in a feasibility trial, where it was deemed feasible, acceptable and potentially efficacious.^10^ The initial version was further revised based on qualitative interviews with prior study participants. POET aims to reduce adolescents’ mental health conditions by reducing the use of maladaptive emotion regulation strategies and increase the use of adaptive ones. Through six modules adolescents are presented with psychoeducation on emotions and skill training in emotion regulation strategies aligned with the Extended Process Model of Emotion Regulation.^18^ Parents participate in an online course to support adolescents in using more adaptive and fewer maladaptive emotion regulation strategies.

### Supportive treatment

An active control treatment enhances internal validity and helps account for nonspecific treatment effects.^21^ When selecting the control condition, methodological rigour, current trial phase and ethical considerations related to participant risk were considered,^22^ as well as disparities in access to, and waiting times for, psychological interventions and challenges in defining treatment-as-usual within primary care in Sweden.^14 23^ Supportive treatment contains no active elements of POET, such as explicit training and therapist support in emotion regulation strategies, treatment goal setting or structured practice. Rather, it is limited to non-specific elements such as self-reflection and general supportive encouragement from the therapist, while being matched to POET in both structure and page count. It has previously been evaluated in clinical trials involving adolescents and their parents^10 24^ and was recently determined to be a credible and feasible control condition to POET.^10^ In the supportive treatment, adolescents are presented with information on mental health and are encouraged to reflect on themes related to well-being such as school, family, self-esteem and peer relationships. For each theme, adolescents will receive information on how these areas may influence mental health and are provided with general advice on how to improve their well-being. Parents participate in an online course including weekly reflections on how to support their adolescent’s well-being.

### Therapists

The therapists delivering the interventions will be licensed clinical psychologists, clinical psychology MSc students or psychotherapists specialised in cognitive behavioural therapy. All therapists will provide both treatments and will receive 1 day training in both methods. Therapists will receive weekly supervision and regular workshops throughout the trial in both treatments. Therapist adherence monitoring is described in the Supplement.

### Outcome measures

Information on the time points for completion of measurements related to all aims are provided in table 1. Details on all measurements, including those related to the additional secondary aims, are described in Supplement.

**Table 1:** Information on the time points for completion and responders of measurements related to all hypotheses

	Trial period										
	Enrolment	Post-randomisation									
Assessment point	Baseline	1w	2w	3w	4w	5w	6w	POST	3M	12M	60M
Enrolment											
Eligibility screen	X										
Informed consent	X										
Randomisation	X										
Treatment POET Supportive treatment		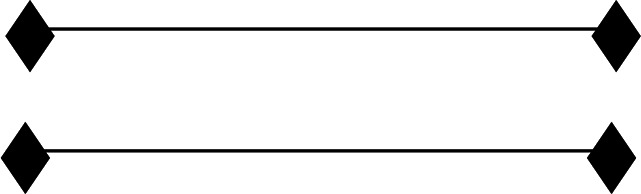									
Assessments											
*Clinician rated*											
MINI-KID	X										
CGI-S	X							X	X	X	X
CGI-I								X	X	X	X
C-GAS	X							X	X	X	X
WSAS	X							X	X	X	X
RCADS-47	X							X	X	X	X
CEQ	X			X							
PIAS				X				X			
*Adolescent rated*											
RCADS-11	X	X	X	X	X	X	X	X	X	X	X
PMERQ-short	X							X	X	X	X
Selected items from PMERQ-short		X	X	X	X	X	X				
DERS-16	X							X	X	X	X
Selected items from DERS-16		X	X	X	X	X	X				
ERQ-CA	X							X	X	X	X
Selected items from ERQ-CA		X	X	X	X	X	X				
PAQ-S	X	X	X	X	X	X	X	X	X	X	X
DSHI-Y									X	X	X
PANAS	X	X	X	X	X	X	X	X	X	X	X
SEQ-C	X	X	X	X	X	X	X	X	X	X	X
BSL-23	X							X	X	X	X
EBQ	X							X	X	X	X
CHU-9D	X							X	X	X	X
CSQ								X			
Study specific questionnaire on Adverse events								X			
CEQ			X								
One selected item from CEQ	X			X	X	X	X				
Single-item assessment of suicidal ideation and plans	X	X	X	X	X	X	X	X	X	X	X
CSQ								X			
*Parent rated*											
RCADS-47	X							X	X	X	X
DERS-16	X							X	X	X	X
CCNES-A	X							X	X	X	X
BERQ	X							X	X	X	X
TIC-P	X							X	X	X	X
CEQ			X								
One selected item from CEQ	X			X	X	X	X				
CSQ								X			

BERQ, The Behavioural Emotion Regulation Questionnaire; BSL-23, Borderline Symptoms List Supplement; CCNES-A, The Coping with Children’s Negative Emotions Scale - Adolescent Version; CEQ, The Credibility/Expectancy Questionnaire; C-GAS, Children’s Global Assessment Scale; CGI-I, The Clinical Global Impressions-Severity; CGI-S, The Clinical Global Impressions-Severity; CHU-9D, The Child Health Utility 9D; CSQ, The Client Satisfaction Questionnaire; DERS-16, The Difficulties in Emotion Regulation Scale - 16-item version; DSHI-Y, Deliberate Self-Harm Inventory Youth version; EBQ, The Emotion Beliefs Questionnaire; ERQ-CA, The Emotion Regulation Questionnaire for Children and Adolescents; 3M, 3 months after treatment; 12M, 12 months after treatment; 60M, 60 months after treatment; MINI-KID, The Mini-International Neuropsychiatric Interview for Children and Adolescents; PANAS, Positive and Negative Affect Schedule for Children; PAQ-S, The Perth Alexithymia Questionnaire-Short Form; PIAS, Internet cognitive–behavioral therapy Adherence Scale; PMERQ, The Process Model of Emotion Regulation Questionnaire – Short; POET, Primary Care Online Emotion Regulation Treatment; POST, immediately after treatment; RCADS-11, The Brief Revised Child Anxiety and Depression Scale for Adolescents; RCADS-47, The Revised Child Anxiety and Depression Scale; SEQ-C, The Social Self-Efficacy subscale of Self-Efficacy Questionnaire; TIC-P, Trimbos Questionnaire for Costs associated with Psychiatric Illness; WSAS, The Work and Social Adjustment Scale.

### Demographic background data

Demographic background data will be collected, including demographics such as parental educational level, occupational status and country of birth, as well as parental psychiatric diagnoses. Adolescents will report on gender identity, assessed inclusively with response options extending beyond the gender binary.

### Primary outcome measure

The primary outcome is clinical global symptom severity, measured with The Clinical CGI-S.^12^ The CGI-S is an ordinal scale ranging from 1 to 7, with higher scores indicating greater severity. CGI-S is administered by a therapist before randomisation and immediately after treatment (primary endpoint), and 3 months after treatment by blinded assessors.

### Secondary outcome measures

Secondary clinician-rated outcome measures include global improvement compared with baseline measured with The CGI-Improvement (CGI-I).^12^ Global and impaired functioning will be measured with CGAS^13^ and The Work and Social Adjustment Scale.^25^ Symptoms of depression and anxiety will be measured with the clinician-rated RCADS-47.^16^ Patient adherence will be assessed by a clinician midway through treatment and immediately after treatment using Internet cognitive–behavioural therapy Adherence Scale.^26^

Secondary adolescent-rated outcomes include facets of emotion regulation which will be measured with a short version of The Process Model of Emotion Regulation Questionnaire^27^ and Difficulties in Emotion Regulation Scale-16 item version.^28^ Additional adolescent-rated outcomes include alexithymia (The Perth Alexithymia Questionnaire-Short Form)^29^ and health-related quality of life (The Child Health Utility 9D.^30^

Parent-rated outcome measures will capture adolescent’s healthcare and other societal resource use, using an adapted version of the Trimbos Questionnaire for Costs associated with Psychiatric Illness (TiC-P).^31^ Parent-related resource use will include productivity losses, specifically absenteeism and presenteeism from work associated with the child’s health. Study-specific parental reports will be used to assess changes in psychotropic medication throughout the study.

### Additional outcomes

Treatment satisfaction will be rated by both adolescents and parents using Client Satisfaction Questionnaire.^32^ Therapist time spent on weekly feedback will be automatically recorded.

### Patient and public involvement

The patient involvement board consists of user representative organisations (Self-Harm and Eating Disorder Organisation [SHEDO] and Attention) as well as adolescents between 12–17 years old. POET was developed in collaboration with the patient involvement board. The board reviewed all treatment modules and provided structured written feedback on the content and relevance. In addition, representatives from user organisations reviewed all participant-related procedures, including the enrolment phase, initial screening, and baseline interviews. They also completed all assessments and provided feedback on the assessment procedures. In addition, during the process of revising and refining POET, interviews were conducted with adolescents and parents participating in the feasibility trial, and their feedback was systematically integrated into the development of the updated treatment version.

### Power analysis

A simulation-based power analysis, based on results from the pilot study, showed that 350 participants would give us>80% power to detect an interaction between treatment and time with an OR of 2.2. To allow for ~10% attrition, we set N to 388 participants. See Supplement for details.

### Statistical analysis

#### Analysis of baseline characteristics

Demographic and baseline characteristics will be summarised for each treatment group. In accordance with Consolidated Standards of Reporting Trials (CONSORT) 2025 recommendations, no significance testing of baseline differences will be performed.^33^ Data distributions and potential outliers will be visually inspected, and sensitivity analyses will be run without outliers. Categorical variables will be summarised as counts and percentages; ordinal and continuous variables as medians and IQRs or means and SD, as appropriate. Data will be analysed according to the participants’ original treatment allocation according to intention-to-treat principles, including all available data in all analyses, with no cases omitted.

#### Primary outcome analysis

To evaluate the primary outcome, change in CGI-S scores will be analysed using a cumulative link mixed model (CLMM) implemented in the ordinal package in R (R Project for Statistical Computing).^34^ The model will include a random intercept for participant, a dummy-coded time variable (before treatment, immediately after treatment and 3 months after treatment, with before treatment as the reference category), the treatment condition, and the interaction between the time variable and treatment. Coefficients from the CLMM represent the log odds of being classified in a higher CGI-S category; these will be converted to ORs.

#### Secondary outcomes analyses

For secondary outcomes, linear mixed-effects regression models, with the same predictors and covariates as the CLMM model will be used. Effect sizes will be estimated as Cohen d for mixed-effects models by dividing the unstandardised β coefficient for the time x treatment interaction by the baseline SD, with 95% bootstrap CIs derived from 1000 simulations. Under the assumption that data are missing at random, mixed-effects regression analyses for repeated measures (including all assessment points) provide unbiased estimates and standard errors.^35^ This assumption indicates that, conditional on observed data, the probability of missingness depends only on observed variables and not on unobserved values.^36^ If >30% of the primary outcome data are missing at the primary end-point, we will conduct NMAR (not missing at random) sensitivity analyses to assess the robustness of the results to departures from the MAR (missing at random) assumption. For CGI-I, the number of participants classified as responders will be presented. Treatment response is defined as a CGI-I rating of 1 (‘very much improved’) or 2 (‘much improved’).^10 37 38^ Differences between treatment groups in participant satisfaction and therapist time will be analysed using independent samples t tests. To examine whether blinded assessors’ guesses of treatment allocation differ from chance, a chi-square test will be conducted. All tests will be two-sided, with statistical significance set at p<0.05.

### Health economic evaluations

The analysis will adhere to the Consolidated Health Economic Evaluation reporting Standards 2022 (CHEERS 2022) statement.^39^ The following analysis will be conducted: a cost-utility analysis using cost per quality-adjusted life year (QALY) gained; and a cost-effectiveness analysis using cost per responder.^40^ Based on previous research,^10 37 38^ treatment response will be defined as a CGI-I rating of 1 (‘very much improved’) or 2 (‘much improved’).^41^ QALYs will be estimated using CHU9D^30^ utility scores, which will be derived using a validated algorithm^42^; and total QALYs over the trial period will be calculated using the area under the curve approach.^43^ Analyses will be conducted from both a healthcare sector and a societal perspective. Total costs will include the costs of administering POET or supportive treatment, as well as the use of other societal resources collected with the TIC-P^31^ including healthcare resources, medication, social and school support services, and productivity losses due to absenteeism and presenteeism related to work. Total costs will be calculated for the full trial period by multiplying frequencies of resources by their respective unit costs. Regression models will be employed to estimate the differences in mean costs and mean effects between the groups over time. Incremental cost-effectiveness ratios will be calculated as the difference in costs between the two interventions divided by the difference in effects (QALYs and response), for each type of analysis and each costing perspective. Sensitivity and scenario analysis will be conducted to explore the uncertainty around the estimates and different assumptions. The uncertainty will be plotted on cost-effectiveness planes, and the probability of the intervention being cost-effective will be calculated across different value levels a decision maker would be willing to pay and plotted on cost-effectiveness acceptability curves.^44^

### Quality control

Although this trial is not a pharmaceutical trial, it will adhere to the main Good Clinical Practice principles. Monitoring procedures are detailed in the Supplement. Data will be collected through a secure online data collection platform operated by the sponsor (ie, Karolinska Institutet). Data will be stored in accordance with the sponsor’s archival policies for research documentation and Swedish legislation.

### Ethics and dissemination

The study has obtained ethical approval from the Swedish Ethical Review Authority (approval no. 2023-03652-01). Prior to the inclusion assessment, both the parent and the adolescent receive age-adapted verbal and written study information, and written electronic consent is collected. Participants can withdraw from the trial at any time. Findings will be disseminated through publication in peer-reviewed journals and presented at scientific conferences.

### Trial status

Participant recruitment began in October 2023 and is anticipated to be completed by February 2026. The last enrolled participant is expected to reach the primary endpoint in April 2026, and the 3-month follow-up in July 2026.

## Discussion

This trial has the potential to generate high-quality evidence on a brief, online, transdiagnostic, theory-based intervention targeting emotion regulation in adolescents with mental health conditions. By targeting emotion regulation, the study may contribute to a better understanding of its role in adolescent mental health. This trial may also inform a scalable and cost-effective approach to delivering evidence-based care in primary care settings. The findings are expected to provide valuable insights for policymakers, healthcare providers and researchers regarding the implementation of psychological treatments for a highly prioritised population.

## Supplementary material

10.1136/bmjopen-2026-116511 online supplemental file 1
