# Supplementary material for: Randomised superiority trial evaluating an online transdiagnostic emotion regulation treatment for adolescents with mental health conditions: study protocol
Source: BMJ Open. 2026 Jul 29;16(7):e116511. doi: 10.1136/bmjopen-2026-116511 (PMC13422934; doi:10.1136/bmjopen-2026-116511)
Supplement: online supplemental file 1 [file bmjopen-16-7-s001.docx]

TRIAL PROTOCOL

A Randomized Superiority Trial Evaluating an Online Transdiagnostic Emotion Regulation Treatment for Adolescents with Mental Health Problems

Table of Content

[Administrative information 4](#_Toc218263717)

[Revision history 5](#_Toc218263718)

[Contact information 6](#_Toc218263719)

[Open science 7](#_Toc218263720)

[Funding/Support: 7](#_Toc218263721)

[Data Sharing Statement: 7](#_Toc218263722)

[Introduction 8](#_Toc218263723)

[Background and rationale 8](#_Toc218263724)

[Purpose and aims 8](#_Toc218263725)

[Background 8](#_Toc218263726)

[Trial objectives 9](#_Toc218263727)

[Primary objective 9](#_Toc218263728)

[Secondary objective(s) 9](#_Toc218263729)

[Research questions and hypotheses 10](#_Toc218263730)

[Primary endpoint 11](#_Toc218263731)

[Secondary endpoint 11](#_Toc218263732)

[Methods: Patient and public involvement, trial design 11](#_Toc218263733)

[Overall trial design 11](#_Toc218263734)

[Patient involvement board 12](#_Toc218263735)

[Methods: participants, interventions, and outcomes 12](#_Toc218263736)

[Procedures 12](#_Toc218263737)

[Start and termination of the trial 13](#_Toc218263738)

[Start of the clinical trial 13](#_Toc218263739)

[End of the clinical trial 13](#_Toc218263740)

[Subject selection 13](#_Toc218263741)

[Inclusion Criteria: 13](#_Toc218263742)

[Exclusion criteria: 13](#_Toc218263743)

[Screening and inclusion 14](#_Toc218263744)

[Withdrawal criteria 14](#_Toc218263745)

[Figure 1. CONSORT Flow Diagram 15](#_Toc218263746)

[Trial treatments 16](#_Toc218263747)

[Treatment format 16](#_Toc218263748)

[Primary Care Online Emotion Regulation Treatment (POET) 16](#_Toc218263749)

[The active control treatment 17](#_Toc218263750)

[Therapists 17](#_Toc218263751)

[Therapist contact 18](#_Toc218263752)

[Treatment adherence monitoring 18](#_Toc218263753)

[Outcome measures 19](#_Toc218263754)

[Primary Outcome Measure 19](#_Toc218263755)

[Secondary Outcome Measures 19](#_Toc218263756)

[Clinician Rated Measures: 19](#_Toc218263757)

[Adolescent Rated Measures 20](#_Toc218263758)

[Parent rated measure 22](#_Toc218263759)

[Adolescent, parent, and clinician measures: 23](#_Toc218263760)

[Distal outcomes 24](#_Toc218263761)

[Table 1. Relevant registers and the types of data to be collected for analyses of distal outcomes 24](#_Toc218263762)

[Table 2. Information on the time points for completion and responders of measurements related to all hypotheses 25](#_Toc218263763)

[Reimbursement 27](#_Toc218263764)

[Inter-Rater Agreement 27](#_Toc218263765)

[Methods: Assignment of interventions 28](#_Toc218263766)

[Randomization 28](#_Toc218263767)

[Blinding 28](#_Toc218263768)

[Methods: Data collection, management, and analysis](#_Toc218263769)

[29](#_Toc218263769)

[Data management 29](#_Toc218263770)

[Archiving 29](#_Toc218263771)

[Interim analysis 29](#_Toc218263772)

[Statistical methods 30](#_Toc218263773)

[Power analysis 30](#_Toc218263774)

[Primary outcome 30](#_Toc218263775)

[Figure 2. visualization of power analysis results for primary outcome 31](#_Toc218263776)

[Secondary continuous outcomes 31](#_Toc218263777)

[Analysis population 31](#_Toc218263778)

[Inter-rater reliability of primary outcome 32](#_Toc218263779)

[Analysis of Baseline Characteristics 32](#_Toc218263780)

[Primary Outcome Analysis 32](#_Toc218263781)

[Secondary Outcomes Analyses 32](#_Toc218263782)

[Health economic evaluation 33](#_Toc218263783)

[Mediation and moderation analyses 34](#_Toc218263784)

[Long-term and distal outcomes data 34](#_Toc218263785)

[Methods: Monitoring 35](#_Toc218263786)

[Trial monitoring 35](#_Toc218263787)

[Quality Control and Quality Assurance 35](#_Toc218263788)

[Ethics 35](#_Toc218263789)

[Benefit-risk evaluation 35](#_Toc218263790)

[Measures to minimize risks for participants 36](#_Toc218263791)

[Handling of Adverse Events 36](#_Toc218263792)

[Definition: Adverse Event (AE) 36](#_Toc218263793)

[Definition: Serious Adverse Event (SAE) 37](#_Toc218263794)

[Assessment of Adverse Events (AE) 37](#_Toc218263795)

[Assessment of causal relationship 37](#_Toc218263796)

[Assessment of expected AE 38](#_Toc218263797)

[Assessment of intensity 38](#_Toc218263798)

[**Table 3.** Content of the adolescent program in POET Treatment 40](#_Toc218263799)

[**Table 4.** Content of the parent course in POET Treatment 41](#_Toc218263800)

[**Table 5.** Content of the adolescent program in Supportive Treatment 42](#_Toc218263801)

[**Table 6.** Content of the parent course in Supportive Treatment 43](#_Toc218263802)

[References 44](#_Toc218263803)

# Administrative information

| Trial ID: | ClinicalTrials.gov identifier: [NCT06067165](https://clinicaltrials.gov/study/NCT06067165). Date for submission: [2023-09-27](https://clinicaltrials.gov/study/NCT06067165?tab=history&a=1#version-content-panel)  Swedish Ethical Review Authority approval number: 2023-03652-0 |
| --- | --- |
| Version number: | 3 |
| Date: | 06-11-2025 |
| Sponsor: | Karolinska institutet |
| Co-sponsor: | Region Stockholm  Region Skåne  Region Östergötland  Västra götalandsregionen |
| Principal Investigator | Associate Professor Johan Bjureberg |

# Revision history

| **Protocol version** | **Date of issue (yyyy-mm-dd)** | **Summary of changes** |
| --- | --- | --- |
| 3 | 2024-07-02 | Assessment of self-harm was added to the follow-up assessment |
| 2 | 2024-06-12 | The parental demographic questionnaire was updated to clarify the item on ethnicity |

# Contact information

| Principal Investigator Johan Bjureberg | Johan Bjureberg, Associate professor  Karolinska institutet |
| --- | --- |

# Open science

## Funding/Support:

Supported by grants provided by the Kavli Trust, Region Stockholm (NSV project: FoUI-937418, FoUI-990404), and Swedish research council (registration number: 2023-05756). Bjureberg is supported by the Royal Swedish Academy of Letters, History and Antiquities, and Stiftelsen Natur & Kultur. Sjöblom is supported by the Queen Silvia Jubilee Fund Foundation. The funding sources have no role in the design and conduct of the study; collection, management, analysis, and interpretation of the data; preparation, review, or approval of manuscripts.

## Data Sharing Statement:

Statistical analysis code will be deposited in the Open Science Framework and KI Data Repository. Due to regulations concerning personal data, the study data is not publicly available. Patient-level data are not publicly available due to national (Swedish) and EU legislation but could be made available from the corresponding author upon reasonable request following approval from the Swedish Ethical Review Authority.

# Introduction

## Background and rationale

### Purpose and aims

The overall purpose of the project is to build an evidence base for a highly scalable transdiagnostic intervention called Primary care Online Emotion-regulation Treatment (POET) for youth seeking treatment for mental health problems.

### Background

Mental health problems in youth are a global problem, causing incalculable suffering in youth and families, harming long-term prospects of youths, and creating substantial economic costs to society. Although treatments exist, these treatments are not fully addressing these problems for several reasons. First, although many youth in need of treatment for mental health problems do not meet criteria for any mental health disorder^1–3^ or they meet criteria for several disorders^4^, most treatments focus on a subset of mental disorders and do not address subthreshold or multi-disorder mental health problems. Second, available treatments do not typically target transdiagnostic disease mechanisms. Third, the available treatments are limited in efficacy.^5^ Fourth, most youth in need do not receive treatment due to social stigma and structural barriers such as geographical distance to treatment providers.^6^ Of particular concern is the lack of attention to subthreshold or multi-disorder mental health problems.^2,4^There is a lack of evidence-based treatment protocols for patients without a formal disorder or fulfilling criteria for several disorders – a problem that has recently been identified within Swedish primary mental health care.^7^ Many leading researchers and institutions have suggested that progress within mental health research and treatment requires a shift away from a focus on specific mental disorders to a more transdiagnostic perspective, addressing underlying processes.^8^ One promising observation in this direction is the robust finding that the frequency of usage of adaptive and maladaptive emotion regulation strategies is related to a wide range of mental health problems, suggesting that emotion regulation may be an important treatment target.^9^ Indeed, a meta-analysis indicates that interventions targeting emotion regulation in youth can improve mental health problems.^10^ However, the authors highlight that there is limited evidence with respect to interventions targeting emotion regulation in different age groups and psychopathologies, as well as a lack of longitudinal studies investigating the temporal relationship between emotion regulation and psychopathology. The authors conclude that appropriately sized randomized controlled trials (RCT) evaluating scalable transdiagnostic treatments targeting emotion regulation are urgently needed to assess both how well they work in youth (i.e., improve mental health) and the mechanisms by which they work (e.g., through improved emotion regulation).

## Trial objectives

In a RCT, we will examine the effects of POET immediately after treatment and at 3-month, 1-year, and 5-year follow-ups on mental health problems and emotion regulation. Secondarily, we will examine if POET is cost-effective, if emotion regulation mediates reduction in mental health problems during treatment, and if POET is more effective for some individuals than others. We will also test whether there are detectable effects of POET on distal outcomes utilizing registry data following participants up to 10 years post treatment.

### Primary objective

The primary objective is to evaluate whether a brief, online, transdiagnostic emotion regulation treatment is more effective than an active control condition in reducing clinical global symptom severity immediately after treatment.

### Secondary objective(s)

Secondary objectives include examining durability of treatment effects on clinical global symptom severity, and treatment effects on emotion regulation, mental health symptoms and, functioning as well as conducting a health economic evaluation.

Additional secondary aims include a mediation analysis to test whether improvements in emotion regulation mediate treatment effects, moderation analyses, and assessments of the durability of treatment effects and other distal outcomes.

## Research questions and hypotheses

1. What are the effects of POET on mental health problems (clinical global symptom severity, mental health symptoms and functioning)?

Hypothesis 1.1: Compared to the active control condition, POET will result in greater reductions in clinical global symptom severity and mental health symptoms, and improvement in functioning immediately after treatment (primary endpoint) and 3-months after treatment.

1. What are the effects of POET on emotion regulation?

Hypothesis 2.1: Compared to the active control condition, POET will result in improved emotion regulation (i.e., reduced maladaptive and enhanced adaptive emotion regulation immediately after treatment and 3-months after treatment.

1. Are reductions in mental health problems during POET treatment mediated through emotion regulation?

Hypothesis 3.1: It is hypothesized that improvement in emotion regulation will temporally precede and mediate reductions in mental health problems during treatment.

1. Is POET cost-effective

Hypothesis 4.1: It is hypothesized that POET will be cost-effective compared to the active control condition

1. Does POET work better for some individuals than others (i.e., moderation)

Hypothesis 5.1: Individual differences in baseline levels of emotion regulation and mental health problems will moderate treatment outcome.

1. Are there detectable differences in the durability of treatment effects and other distal outcomes between POET and the active control at 1-year, 5-year and 10-year after treatment, and do individual baseline characteristics moderate these effects?

Hypothesis 6.1: Compared to the active control condition, POET is expected to produce greater treatment effects, including reductions in clinical global symptom severity improved emotion regulation ability, reduced maladaptive and enhanced adaptive emotion regulation in the long term, better subsequent academic performance, fewer mental health disorders and self-harm behaviors, and reduced healthcare utilization and consumption of medication for psychiatric symptoms.

## Primary endpoint

The primary endpoint of this trial is immediately after treatment (7 weeks after receiving the first module)

## Secondary endpoint

Secondary endpoints are:

- 3 months after treatment.
- 1 years after treatment.
- 5 years after treatment.
- Distal outcomes will be examined after the participant’s 18th birthday using data from Swedish national registries retrieved 1 to 10 years after treatment completion, depending on age at study inclusion.
- Weekly during treatment.

# Methods: Patient and public involvement, trial design

## Overall trial design

This study adopts a RCT design. The study has an active control treatment. The study will be hosted at different well-established youth primary care services in Sweden. Participants are assessed before treatment and then weekly during treatment. Subsequently, follow-up assessments are completed; immediately after treatment, 3 months after treatment, 1-year, 5-year and 10-year after treatment. 388 adolescents (ages 12-17 years) with mild- moderate mental health problems will be randomized to one of two treatment conditions:

a) Online emotion regulation treatment (n = 194)

b) Online supportive treatment (n = 194).

## Patient involvement board

The patient involvement board consists of user representative organizations ([SHEDO](https://www.shedo.se/) and [Attention](https://attention.se/)) as well as adolescents between 12–17 years old. POET was developed in collaboration with the patient involvement board. The board reviewed all treatment modules and provided structured written feedback on the content and relevance. In addition, representatives from user organizations reviewed all participant-related procedures, including the enrollment phase, initial screening, and baseline interviews. They also completed all assessments and provided feedback on the assessment procedures. In addition, during the process of revising and refining POET, interviews were conducted with adolescents and parents participating in the feasibility trial, and their feedback was systematically integrated into the development of the updated treatment version.

# Methods: participants, interventions, and outcomes

## Procedures

Participants will be recruited nationally. Information about the research project will be disseminated to primary care and first line mental health clinics and through advertisements on social media. Potential participants register via the project's website ([www.poetstudien.se](http://www.poetstudien.se)) where they receive age-appropriate information about the study.

## Start and termination of the trial

### Start of the clinical trial

The start of the trial is defined as when the first participant is randomized

### End of the clinical trial

The trial ends when the final registry data have been retrieved.

## Subject selection

### Inclusion Criteria:

- Age of 12 to 17 years (under 18).
- Presence of mental health problems (defined as a Clinical Global Impressions–Severity (CGI-S) score of 2 or higher.
- Having at least 1 parent willing to participate in the parent course.

### Exclusion criteria:

- Severe mental illness requiring specialized care or low global functioning corresponding to a Children’s Global Assessment Scale (CGAS) score of less than 41 on a scale of 1 to 100 (higher scores indicating better functioning).
- Acute suicidality, (ongoing suicide plans or recent suicide attempt).
- Ongoing psychological treatment.
- Changes in psychopharmacological medication during the past 2 months.
- Insufficient Swedish comprehension.
- Life circumstances that could prevent treatment participation acute suicidality, (ongoing suicide plans or recent suicide attempt).

### Screening and inclusion

Subject eligibility (that subjects fulfil all inclusion criteria and do not meet any exclusion criteria) is established before randomization. Participants can be referred by other treatment provider or make a self-referral via the self-assessment tool and website called BASS (centrally administered by the sponsor Karolinska Institutet). When potential participants have been referred to the study or have made a self-referral, a therapist (psychologist, psychotherapist or clinical psychology M.Sc. student under supervision) will contact the guardian for an initial telephone screening to evaluate whether the youth fills inclusion and not exclusion criteria. The therapist will explain the study procedure and any questions regarding the procedure will be answered. This initial screening is estimated to take about 15 minutes. If still interested, the potential participant and guardian is invited to a digital video-call clinical assessment. Before this assessment, the guardian and adolescent receive login information to the self- assessment tool BASS, where the research person information, self- assessment measures and informed consent registration are available. Guardians and adolescents answer self-assessment measures and informed consent is collected. The part of the study concerning distal outcomes is optional. If participants choose not to consent to the retrieval of registry data, this will not affect their participation in the study or the treatment they receive. Potential participants and parents completed an assessment interview over video link, including a semi structured interview,^11^ and clinician-assessed forms. Any questions that the participant or guardians have regarding the procedure is answered. Individuals who meet the inclusion criteria and no exclusion criteria are offered participation in the study. If needed, excluded participants receive recommendations for other treatment.

### Withdrawal criteria

Subjects can discontinue their participation in the trial at any time without any consequence to his/her continued treatment. If the subject discontinues the trial, follow-up of this subject will be performed according to the clinic’s routine.

# Figure 1. CONSORT Flow Diagram

5 years after treatment

Intention-to-treat analysis

1 year after treatment

Intention-to-treat analysis

3 months after treatment

Intention-to-treat analysis

5 years after treatment

Intention-to-treat analysis

Immediately after treatment

Intention-to-treat analysis

Allocated to POET

Received allocated intervention

Did not receive allocated intervention

Immediately after treatment

Intention-to-treat analysis

3 months after treatment

Intention-to-treat analysis

1 year after treatment

Intention-to-treat analysis

Allocated to Supportive treatment

Received allocated intervention

Did not receive allocated intervention

Excluded

Not meeting inclusion criteria

Declined to participate

Other reasons

Randomized

Allocation

Enrolment

Assessed for eligibility

##

# Trial treatments

### Treatment format

Treatments and parent course in both conditions will be delivered in a blended treatment format combining asynchronous therapist-guided online modules (text/videos/audio/messaging function) with synchronous sessions delivered over video-link or live at treatment week 0, 2 and 6. The first digital session is designed to introduce participants and their parents to the treatment platform and to establish a structured plan for when the family will engage with the treatment material. The second digital session focuses on identifying and addressing potential motivational or practical barriers to participation. In the POET condition, adolescents´ vulnerability and resilience are addressed and treatment goals are formulated (Specific, Measurable, Achievable, Relevant, and Time-bound goals) collaboratively with the family in accordance, whereas no individualized goal setting is conducted in the active control condition. The final digital session aims to summarize the treatment period and assess the potential need for continued care.

### Primary Care Online Emotion Regulation Treatment (POET)

POET is theoretically grounded in the Extended Process Model of Emotion Regulation, which outlines the emotion-generative sequence and four families of regulation strategies: situational, attentional, cognitive, and response modulation.^12,13^ Further, POET has been adapted from online Emotion Regulation Individual Therapy for Adolescents (IERITA) for youth with nonsuicidal self-injury.^14^ An initial version of the POET treatment was evaluated in a feasibility trial, where it was deemed acceptable and potentially efficacious.^15^ The initial version was further revised based on qualitative interviews with prior study participants. POET aims to reduce adolescents´ maladaptive emotion regulation strategies, enhancing adaptive emotion regulation strategies and abilities and thereby, reduce mental health problems. Through 6 modules adolescents are presented with psychoeducation on emotions and skill training in emotion regulation strategies aligned with the Extended Process Model of Emotion Regulation.^13^ The first module introduces the treatment rationale. Module 2 focuses on reducing avoidance, confronting unpleasant situations, and seeking out positive experiences. Module 3 aims to increase awareness and focus on one’s emotions. Module 4 addresses negative beliefs about emotions and emotion regulation, promotes mindful acceptance of thoughts, and encourages the development of helpful appraisal styles. Module 5 focuses on reducing emotional suppression and replacing emotion-driven behavioral impulses with more adaptive responses. Finally, Module 6 provides repetition and a summary of the key concepts and skills covered throughout the treatment. The parent component will include psychoeducation and teaching effective support and responding to their children’s and their own emotions. Detailed descriptions of the treatment and the parent course are provided in Table 3 and Table 4.

### The active control treatment

The active control will be Supportive treatment, a stringent comparator previously evaluated by members of our research group in an online treatment study for social anxiety disorder in youths.^16^ The active control will be designed to control for effects of non-specific treatment components that are common to most psychosocial interventions, such as receiving counseling, attention from a therapist, and monitoring of one’s emotions and behaviors. The control treatment mimicked supportive therapy, a well-established comparator in adolescent clinical trials.^17^ It included no active elements of POET; rather, it consisted of information on mental health, and participants were encouraged to reflect on themes related to well-being (e.g., school and friends).^16^

Parents enrolled in an online course including weekly reflections on how to support their adolescent’s well-being. The active control treatment showed high acceptability and utility in previous pilot study. Detailed descriptions of the active control treatment and the parent course are provided in Table 5 and Table 6.

### Therapists

The therapists delivering the interventions are licensed clinical psychologists, clinical psychology M.Sc. student under supervision or psychotherapists specialized in cognitive behavioral therapy. Therapists will provide both interventions, after one day training in both methods. Therapists will receive on demand and weekly supervision throughout the trial in addition to regular workshops in both treatments. The same therapists provide both POET and the control treatment, after careful training in both methods. The purpose of this is to decrease the probability of confounding of therapists’ characteristics and the intervention.

### Therapist contact

Therapists will have weekly contact with participants via a secure web platform. Participants and guardians will report weekly on their home-assignments and can also submit questions and will receive feedback on their reports and questions within two working days. At treatment week 0, 2 and 6 a digital meeting between therapist and participant is held. All digital meetings will be audio recorded to enable assessment of treatment protocol adherence.

### Treatment adherence monitoring

Ten percent of all synchronous video meetings conducted after the first module (audio recordings) and therapist feedback messages (text) will be rated for adherence in both treatment conditions.

Two raters both licensed clinical psychologists with experience in the POET treatment and internet-delivered interventions for children, will perform the ratings. The first three cases were double rated by both raters, with no discrepancies observed.

Participant IDs (numbers between 1 and 10) and module numbers are randomly selected using a digital randomization tool (random.org). One participant is selected from each set of ten participants, and once a participant has been rated, they will not be included in subsequent ratings. All therapists are rated at least once per treatment condition.

Adherence ratings of therapist contact and feedback to both adolescents and parents are based on the Adherence Scale for Internet-Based Treatment.^18^ The rating procedure also includes study-specific prohibited elements and clarifications of desirable therapist behaviors within the POET framework.

The rating of synchronous video meetings after the first module is based on the structured Check-in interview template, along with a list of pre-specified prohibited elements in both treatments. The template was pilot tested on the first rated participant and subsequently revised. Ratings cover the entire contact, including interactions with both the adolescent and the parent.

# Outcome measures

## Primary Outcome Measure

**Clinician rated clinical global symptom severity** will be measured with The Clinical Global Impressions-Severity (CGI-S).^19^The CGI-S is an ordinal scale ranging from 1 to 7, with higher scores indicating greater severity. CGI-S is administered by a blinded assessor before randomization and at immediately after treatment (primary endpoint), 3 months after treatment, and at 1 and 5 years follow.

## Secondary Outcome Measures

### Clinician Rated Measures:

**Global improvement** will be assessed by a blinded assessor using CGI- Improvement (CGI-I).^19^ The CGI-I is a single-item measure that rates overall symptom change compared to baseline on a 7-point scale, where lower scores indicate greater improvement.

**Global and impaired functioning** will be assessed using the clinician-rated Children’s Global Assessment Scale (CGAS).^20^ The CGAS is a single-item measure ranging from 1 to 100, with higher scores indicating better overall functioning.

**Functional impairment** across five domains: school, everyday activities, friendships and social life, recreation and hobbies, and family or close relationships will be measured using The Work and Social Adjustment Scale (WSAS)^21^will be used to assess. The scale consists of five items, yielding a total score ranging from 0 to 40, with higher scores indicating greater impairment.

**Symptoms of depression and anxiety** will be assessed using The Revised Child Anxiety and Depression Scale (RCADS-47).^22^ RCADS-47 measures symptoms of both anxiety and depression, yielding a total score ranging from 0 to 141, with higher scores indicating greater symptom severity.

**Clinician predictions** of treatment response will be collected prior to randomization. Clinicians will be asked to make an overall assessment based on all available information about the participant and estimate the most likely outcome on the CGI-S/I at the end of treatment.

**Therapist time:** The total therapist time spent on both reading what adolescents and parents have written in the modules and providing feedback and messaging participants will be calculated.

**Patient adherence** will be assessed by a clinician midway through treatment using Internet Cognitive Behavior Therapy Adherence Scale (PIAS).^23^

### Adolescent Rated Measures

**Symptoms of depression and anxiety** will be assessed using The Brief Revised Child Anxiety and Depression Scale for Adolescents (RCADS-11)^24^ will be completed by adolescents to assess self-reported symptoms of depression and anxiety. This version consists of 11 items, producing a total score ranging from 0 to 33, with higher scores indicating more severe symptoms.

**Individual differences in emotion regulation** will be measured using a short version (developed for the purpose of this study) of The Process Model of Emotion Regulation Questionnaire - Short (PMERQ).^25^ The scale consists of 30 items, yielding a total score ranging from 30 to 180, with higher scores indicating greater use of emotion regulation strategies. The questionnaire is self-rated by adolescents. Three subscales; Confront Unpleasant Situations (3 items; total score range 6-18), Avoid Unpleasant Situations (3 items; total score range 6-18), and Cognitively Distract (3 items; total score range 6-18) will be administered to adolescents on a weekly basis.

**Difficulties in emotion regulation** will be measured using The Difficulties in Emotion Regulation Scale - 16-item version (DERS-16).^26^ The scale consists of 16 items, yielding a total score ranging from 16 to 80, with higher scores indicating greater difficulties in emotion regulation. The questionnaire is self-rated by adolescents. The DERS-16 is additionally administered to parents to measure their self-reported difficulties in emotion regulation. The Impulse subscale (3 items; total score range 3–15) will be administered weekly during treatment.

**Cognitive reappraisal** will be measured with one subscale of The Emotion Regulation Questionnaire for Children and Adolescents (ERQ-CA).^27^ The cognitive reappraisal subscale consists of 6 items, yielding a total score ranging from 6 to 30, with higher scores indicating greater use of cognitive reappraisal strategies. The questionnaire is self-rated by adolescents.

**Levels of alexithymia** will be measured using The Perth Alexithymia Questionnaire – Short Form (PAQ-S).^28^ The scale consists of 6 items, yielding a total score ranging from 6 to 42, with higher scores indicating greater levels of alexithymia.

**Non-suicidal self-injury (NSSI)** during the past month will be assessed by a clinician prior to randomization, using a single clinician-administered item from the Mini International Neuropsychiatric Interview for Children and Adolescent*s* (MINI-KID).^11^

**Self-harm** will be measured using Deliberate Self-Harm Inventory - Youth Version DSHI-Y).^29^ DSHI-Y measures the occurrence, methods, and frequency of deliberate self-harm. The scale consists of 6 items, with higher scores indicating more frequent engagement in self-harm behaviors.

**Positive and negative affect** will be measured using the *Positive and Negative Affect Schedule for Children* (PANAS).^30^ The scale includes two subscales, each with a total score ranging from 5 to 25, where higher scores indicate greater levels of the corresponding affect (positive or negative).

**Self-efficacy** will be measured using the Social Self-Efficacy subscale of Self-Efficacy Questionnaire for Children (SEQ-C).^31^ The Social Self-Efficacy subscale consists of 8 items, yielding a total score ranging from 8 to 40, with higher scores indicating greater perceived social self-efficacy.

**High-risk and problematic behaviors** will be measured using *Borderline Symptoms List Supplement* (BSL-23).^32^ BSL- 23 assess engagement in high-risk and maladaptive behaviors. The scale consists of 10 items, yielding a total score ranging from 0 to 40, with higher scores indicating greater frequency of such behaviors. This version is study-specific and adapted for use in primary care.

**Beliefs about emotions** will be measured using Emotion Beliefs Questionnaire (EBQ).^33^ The scale consists of 16 items, yielding a total score ranging from 16 to 112, with higher scores indicating more maladaptive beliefs about emotions.

**Health-related quality of life** will be measured using the Child Health Utility 9D (CHU-9D).^34^ The CHU-9D consists of 9 items assessing different aspects of health-related quality of life, with a total score ranging from 9 to 45. Higher scores indicate poorer quality of life. CHU-9D has a validated algorithm for calculating QALY in children and adolescents.

**Adverse events:** Participants are asked to report and rate the discomfort of the eventual adverse events caused by their participation in the treatment. These questions will be administered to all adolescents at immediately after treatment.

### Parent rated measure

**Parents’ perceived ability to cope with children’s negative emotions** will be measured using the Coping with Children’s Negative Emotions Scale – Adolescent Version (CCNES-A).^35^ This scale assesses parents’ perceived ability to respond to their children’s negative emotions across six subscales: emotion-focused, problem-focused, minimization, punitive, expressive encouragement, and distress responses. Each subscale includes 9 items, rated on a 7-point scale, with higher scores indicating greater use of that particular coping style, except for the expressive encouragement subscale which is reverse-scored.

**Parents’ perception of adolescents’ behavioral emotion regulation** will be measured using the Behavioral Emotion Regulation Questionnaire (BERQ).^36^ This scale assesses parents’ perceptions of their adolescent’s use of behavioral emotion regulation strategies across five subscales. Each subscale includes 4 items, yielding a total subscale score ranging from 4 to 20, with higher scores indicating greater use of that specific strategy.

**Healthcare and other societal resource use** for both children and caregivers will be reported by parents using the Trimbos/iMTA Questionnaire for Costs associated with Psychiatric Illness (TiC-P).^37^ This instrument assesses healthcare utilization and other societal resource use related to psychiatric illness.

**Demographic background data:** Parents will answer questions about the parental educational level, occupational status, prior treatment history, and information on how the participants got in contact with the study will be registered (e.g., via self-referral or referral from health care).

**Study-specific parental reports** will be used to assess any changes in the child’s psychotropic medication since the end of the POET treatment, including dosage adjustments, discontinuation, or initiation of new medications.

### Adolescent, parent, and clinician measures:

**Credibility and expectancy** will be measured using The Credibility/Expectancy Questionnaire (CEQ).^38^ The scale consists of 6 items which are rated on a 9-point scale (1 = not at all to 9 = very much), except for items 4 and 6, which are rated from 0% to 100%. Higher scores indicate greater perceived credibility and expectancy. The questionnaire is clinician-, adolescent-, and caregiver-reported. Adolescents and parents will complete the full CEQ before treatment and after the first treatment module. Thereafter, one selected CEQ item will be administered weekly to monitor changes in credibility and expectancy over time. Clinicians will complete the CEQ before treatment and after half of the treatment has been completed.

**Client satisfaction is measured using** The Client Satisfaction Questionnaire (CSQ)^39^ will be used to assess satisfaction with treatment. The scale consists of 8 items, yielding a total score ranging from 8 to 32, with higher scores indicating greater treatment satisfaction. The questionnaire is self-rated immediately after treatment by adolescents and caregiver reported.

### Distal outcomes

**Information on other distal outcomes** will be collected through linkage with Swedish national registries. Data will include academic performance (grade point average and eligibility for further education), occupation (employment status, sick leave, and highest level of education), psychopharmacological treatment (prescription and use of medication for psychiatric symptoms), and health care utilization (diagnoses, type of care, and occurrences of self-harm. The relevant registers and the types of data to be collected are presented in the table below.

# Table 1. Relevant registers and the types of data to be collected for analyses of distal outcomes

| **Register** | **Data of interest** |
| --- | --- |
| National Patient Register | Diagnosis, healthcare utilization, type of care, and suicide attempts |
| VAL Database | Diagnosis, healthcare utilization, type of care, and suicide attempts |
| Prescribed Drug Register | Prescription and consumption of medication for psychiatric symptoms |
| LISA (Longitudinal Integration Database for Health Insurance and Labour Market Studies) | Employment status, sick leave, and highest educational level |
| Swedish National Agency for Education: Quality Declaration, Compulsory School (final grades) | Grades and eligibility |
| Swedish National Agency for Education: Quality Declaration, Upper Secondary School (grade records) | Grades and eligibility |

# Table 2. Information on the time points for completion and responders of measurements related to all hypotheses

|  | TRIAL PERIOD | | | | | | | | | | | | |
| --- | --- | --- | --- | --- | --- | --- | --- | --- | --- | --- | --- | --- | --- |
|  | Enrolment | Randomization | Post-randomization | | | | | | | | | | |
| Assessment point | Baseline | | 1w | 2w | 3w | 4w | 5w | 6w | POST | 3M | 12M | | 60M |
| **ENROLMENT** |  | |  |  |  |  |  |  |  |  |  | |  |
| Eligibility screen | X | |  |  |  |  |  |  |  |  |  | |  |
| Informed consent | X | |  |  |  |  |  |  |  |  |  | |  |
| Randomization | X | |  |  |  |  |  |  |  |  |  | |  |
| **TREATMENT**  POET  Supportive treatment |  | |  | | | | | |  | | | | |
| **ASSESSMENTS** |  | |  |  |  |  |  |  |  |  | |  |  |
| ***Clinician rated*** |  | |  |  |  |  |  |  |  |  | |  |  |
| MINI-KID | X | |  |  |  |  |  |  |  |  | |  |  |
| CGI-S | X | |  |  |  |  |  |  | X | X | | X | X |
| CGI-I |  | |  |  |  |  |  |  | X | X | | X | X |
| C-GAS | X | |  |  |  |  |  |  | X | X | | X | X |
| WSAS | X | |  |  |  |  |  |  | X | X | | X | X |
| RCADS-47 | X | |  |  |  |  |  |  | X | X | | X | X |
| CEQ | X | |  |  | X |  |  |  |  |  | |  |  |
| PIAS |  | |  |  | X |  |  |  | X |  | |  |  |
| ***Adolescent rated*** |  | |  |  |  |  |  |  |  |  | |  |  |
| RCADS-11 | X | | X | X | X | X | X | X | X | X | | X | X |
| PMERQ-short | X | |  |  |  |  |  |  | X | X | | X | X |
| Selected items from PMERQ-short |  | | X | X | X | X | X | X |  |  | |  |  |
| DERS-16 | X | |  |  |  |  |  |  | X | X | | X | X |
| Selected items from DERS-16 |  | | X | X | X | X | X | X |  |  | |  |  |
| ERQ-CA | X | |  |  |  |  |  |  | X | X | | X | X |
| Selected items from ERQ-CA |  | | X | X | X | X | X | X |  |  | |  |  |
| PAQ-S | X | |  |  |  |  |  |  |  | X | | X | X |
| DSHI-Y |  | |  |  |  |  |  |  | X | X | | X | X |
| PANAS | X | | X | X | X | X | X | X | X | X | | X | X |
| SEQ-C | X | | X | X | X | X | X | X | X | X | | X | X |
| BSL-23 | X | |  |  |  |  |  |  | X | X | | X | X |
| EBQ | X | |  |  |  |  |  |  | X | X | | X | X |
| CHU-9D | X | |  |  |  |  |  |  | X | X | | X | X |
| CSQ |  | |  |  |  |  |  |  | X |  | |  |  |
| Study specific questionnaire on Adverse events |  | |  |  |  |  |  |  | X |  | |  |  |
| CEQ | X | |  |  |  |  |  |  |  |  | |  |  |
| One selected item from CEQ |  | | X | X | X | X | X | X |  |  | |  |  |
| Single-item assessment of suicidal ideation and plans | X | | X | X | X | X | X | X | X | X | | X | X |
| CSQ |  | |  |  |  |  |  |  | X |  | |  |  |
| ***Parent rated*** |  | |  |  |  |  |  |  |  |  | |  |  |
| RCADS-47 | X | |  |  |  |  |  |  |  | X | | X | X |
| DERS-16 | X | |  |  |  |  |  |  |  | X | | X | X |
| CCNES-A | X | |  |  |  |  |  |  |  | X | | X | X |
| BERQ | X | |  |  |  |  |  |  |  | X | | X | X |
| TIC -P | X | |  |  |  |  |  |  |  | X | | X | X |
| CEQ | X | |  |  |  |  |  |  |  |  | |  |  |
| One selected item from CEQ |  | |  | X | X | X | X | X |  |  | |  |  |
| CSQ |  | |  |  |  |  |  |  |  | X | |  |  |

Abbreviations: 1w, 1 week; 2w, 2 week; 3w, 3 week; 4w, 4 week; 5w, 5 week; 6w, 6 week, POST, Immediately after treatment; 3M, 3 months after treatment; POET, Primary Care Online Emotion Regulation Treatment; MINI-KID,The Mini-International Neuropsychiatric Interview for Children and Adolescents; CGI-S, The Clinical Global Impressions-Severity; CGI-I, The Clinical Global Impressions-Severity; C-GAS, Children’s Global Assessment Scale; WSAS, The Work and Social Adjustment Scale; RCADS-47, The Revised Child Anxiety and Depression Scale; CEQ, The Credibility/Expectancy Questionnaire; PIAS, Internet Cognitive Behavior Therapy Adherence Scale; RCADS-11, The Brief Revised Child Anxiety and Depression Scale for Adolescents; PMERQ, The Process Model of Emotion Regulation Questionnaire – Short; DERS-16, The Difficulties in Emotion Regulation Scale - 16-item version; ERQ-CA, The Emotion Regulation Questionnaire for Children and Adolescents; PAQ-S,The Perth Alexithymia Questionnaire-Short Form; DSHI-Y, Deliberate Self-Harm Inventory Youth version; PANAS, Positive and Negative Affect Schedule for Children; SEQ-C,The Social Self-Efficacy subscale of Self-Efficacy Questionnaire; BSL-23, Borderline Symptoms List Supplement; EBQ, The Emotion Beliefs Questionnaire; CHU-9D, The Child Health Utility 9D; CSQ, The Client Satisfaction Questionnaire, CCNES-A, The Coping with Children’s Negative Emotions Scale - Adolescent Version; BERQ, The Behavioral Emotion Regulation Questionnaire; TIC-P, Trimbos Questionnaire for Costs associated with Psychiatric Illness.

## Reimbursement

Adolescents will be compensated with a gift card worth 100 SEK for each completed assessment after the treatment period (up to a maximum total of 800 SEK).

## Inter-Rater Agreement

Through-out the trial therapist and blind assessors attend regularly workshops in which we conduct inter rater agreement of audio recorded blind assessment interviews. To evaluate inter-rater reliability, 10% of all baseline assessment and blinded assessment ratings will be independently rated by an assessor. The additional rating will be used to estimate the level of agreement between evaluators and to ensure consistency and reliability in clinical assessments across raters. Before conducting assessments all therapist and blind assessors had to conduct inter- rater agreement introduction consisting of several inter-rater agreement ratings. Therapists and blind assessors could not begin conducting ratings until inter-rater agreement had been reached with the other assessors.

# Methods: Assignment of interventions

## Randomization

Eligible participants will be randomly assigned (1:1) to either the POET intervention or the Supportive Treatment condition. Randomization will be conducted in blocks of four or six within each participating Region. An independent researcher, not involved in participant recruitment or treatment delivery, will perform the randomization using an online system (Sealed Envelope Ltd) that employs a secure pseudo-random number generator.

## Blinding

Primary and secondary clinician outcome measures will be assessed by a clinician blind to treatment allocation before treatment (before randomization), immediately after treatment, and 3 months after treatment, and at 1 and 5 years follow up. To ensure the integrity of blinding procedures, participants will be provided with explicit instructions not to reveal their treatment allocation to the blind rater. After completing the assessment, the blind raters guess the participant’s group allocation and disclose the reason for their guess (e.g., true random guess) and report any disclosed group allocation. We will examine whether blinded assessors’ treatment allocation guesses differ from chance. Blind assessments will be audio-recorded to allow for calculation of inter-rater reliability. The participants, therapists, and the project manager are not blind to treatment allocation. Blinded assessors will receive thorough training in the clinician-rated measures and must demonstrate adequate inter-rater agreement before conducting blinded assessments.

## Data management

Subjects who participate in the trial are coded with a trial-specific identification code. All subjects are registered in a subject identification code list that connects the subject’s name and personal number with a subject number/trial identification number. The code key is stored in a security-classified database for sensitive personal data within the organization of the sponsor. Data is stored in accordance with the sponsors archival rules for research documents.

All individuals handling personal data are bound by confidentiality agreements and/or professional secrecy obligations through their employment terms or confidentiality agreements in accordance with the sponsor.

All correspondence between participants and therapists will take place on platforms which require two-factor authentication.

Assessments are administered via the internet and all answers, from participants and clinicians, are securely stored on a database developed for this purpose and have been used in several completed and ongoing studies. All data traffic is encrypted, ensuring a high security level.

### Archiving

Data is archived in accordance with the sponsors archival rules for research documents

### Interim analysis

No interim analysis will be conducted.

## Statistical methods

### Power analysis

#### Primary outcome

A simulation-based power analysis was performed. Based on the data from the pilot study)^15^, a simpler model without random effects was fitted to estimate the effect of time and the thresholds. Data was then generated using these values, and parameters that were varied, namely sample size, interaction between time and treatment (log-odds), and the variance of the random effect. Sample size took the values of 250, 300, 350, and 400, the interaction effect took the values of 0.59, 0.69, 0.79, and 0.88 (corresponding to ORs of 1.8, 2.0, 2.2, and 2.4), and the variance of the random effect took the values of 1.48 and 2.22 (corresponding to ICC of 0.4 and 0.6). For each combination of parameters, 700 datasets were simulated, and a cumulative link mixed model (CLMM) was fitted to the data. The p-value of the interaction effect was saved for each simulation, and power was calculated as the proportion of p-values less than 0.05. The CLMM model fitted in each iteration included a random intercept for participant, a dummy-coded time variable (before treatment, after treatment), the treatment condition, and the interaction between the time variable and treatment. The results from the power analysis are shown in Figure 2.

We concluded that 350 participants would give us >80% power to detect an interaction between treatment and time with an OR of 2.2. To allow for ~10% attrition, we set N to 388 participants.

# Figure 2. visualization of power analysis results for primary outcome


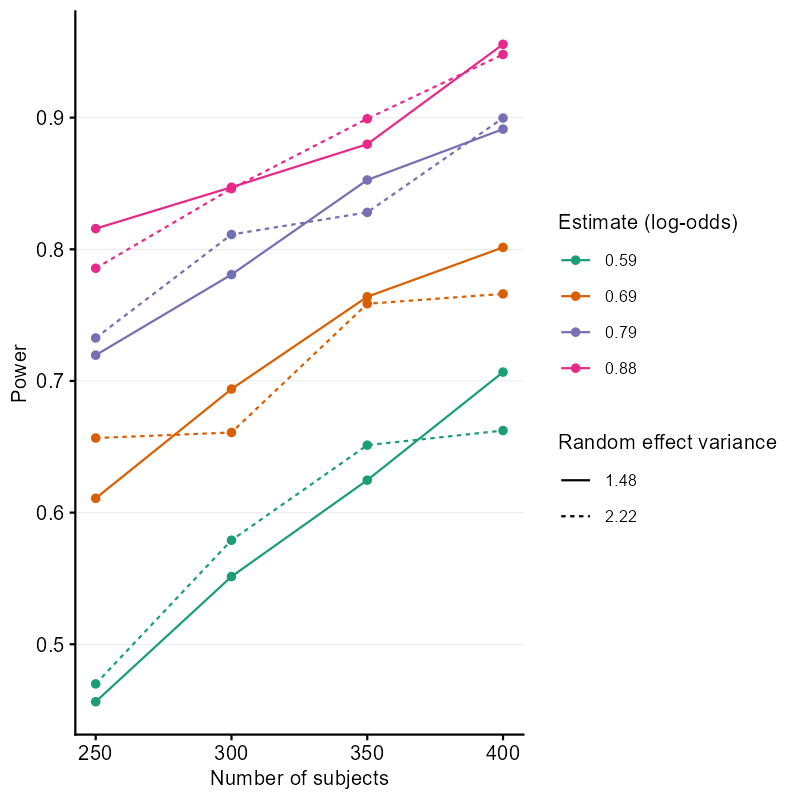


#### Secondary continuous outcomes

A simulation-based power analysis was performed using a linear mixed-effects model. Assuming two time points (before treatment and immediately after treatment), 388 participants (194 per group), 10% attrition, ICC = 0.5, and a two-sided test at α = 0.05, the study has >80% power to detect a difference 0.30 (Cohen’s d) between groups immediately after treatment.

### Analysis population

Analyses will be conducted according to the intention-to-treat (ITT) principle.

### Inter-rater reliability of primary outcome

To assess inter-rater reliability, Krippendorff’s alpha will be calculated, as it is suitable for nominal and ordinal data and allows for different raters to have evaluated different subsets of cases.^40^

### Analysis of Baseline Characteristics

Demographic and baseline characteristics will be summarized for each treatment group. In accordance with CONSORT 2025 recommendations, no significance testing of baseline differences will be performed.^41^ Data distributions and potential outliers will be visually inspected, and sensitivity analyses will be run without outliers. Categorical variables will be summarized as counts and percentages; ordinal and continuous variables as medians and interquartile ranges or means and standard deviations, as appropriate. Data will be analyzed according to the participants’ original treatment allocation in line with intention-to-treat principles, including all available data in all analyses, with no cases omitted.

### Primary Outcome Analysis

To evaluate the primary outcome, change in CGI-S scores will be analyzed using a cumulative link mixed model (CLMM) implemented in the ordinal package in R (R Project for Statistical Computing).^42^ The model will include a random intercept for participant, a dummy-coded site variable, a dummy-coded time variable (before treatment, immediately after treatment, and 3 months after treatment, with before treatment as the reference category), the treatment condition, and the interaction between the time variable and treatment. Coefficients from the CLMM represent the log odds of being classified in a higher CGI-S category, these will be converted to odds ratios.

### Secondary Outcomes Analyses

For secondary outcomes, linear mixed-effects regression models, with the same predictors and covariates as the CLMM model will be used. Effect sizes will be estimated as Cohen d for mixed-effects models by dividing the unstandardized β coefficient for the time x treatment interaction by the baseline SD, with 95% bootstrap confidence intervals derived from 1000 simulations. Under the assumption that data are missing at random, mixed-effects regression analyses for repeated measures (including all assessment points), provide unbiased estimates and standard errors.^43^ This assumption indicates that, conditional on observed data, the probability of missingness depends only on observed variables and not on unobserved values.^44^ If >30% of the primary outcome data are missing at the primary end-point, we will conduct NMAR sensitivity analyses to assess the robustness of the results to departures from the MAR assumption. For CGI-I, the number of participants classified as responders will be presented. Treatment response is defined as a CGI-I rating of 1 (“very much improved”) or 2 (“much improved”).^15,45,46^ Differences between treatment groups in participant satisfaction and therapist time will be analyzed using independent samples t tests. To examine whether blinded assessors’ guesses of treatment allocation differ from chance, a t test will be conducted. All tests will be 2-sided, with statistical significance set at P < .05.

### Health economic evaluation

The analysis will adhere to the Consolidated Health Economic Evaluation reporting Standards 2022 (CHEERS 2022) statement. The following analysis will be conducted: a cost-utility analysis using cost per quality-adjusted life year (QALY) gained; and a cost-effectiveness analysis using cost per responder.^47,48^ Based on previous research,^15,45,46^ treatment response will be defined as a CGI-I rating of 1 (“very much improved”) or 2 (“much improved”). QALYs will be estimated using CHU9D utility scores, which will be derived using a validated algorithm.^49^; and total QALYs over the trial period will be calculated using the area under the curve approach.^50^ Analyses will be conducted from both a health care sector and a societal perspective. Total costs will include the costs of administering POET or supportive treatment, as well as the use of other societal resources collected with the TIC-P, including health care resources, medication, social and school support services, and productivity losses due to absenteeism and presenteeism related to work. Total costs will be calculated for the full trial period by multiplying frequencies of resources by their respective unit costs. Regression models will be employed to estimate the differences in mean costs and mean effects between the groups over time. Incremental cost-effectiveness ratios (ICER) will be calculated as the difference in costs between the two interventions divided by the difference in effects (QALYs and response), for each type of analysis and each costing perspective. Sensitivity and scenario analysis will be conducted to explore the uncertainty around the estimates and different assumptions. The uncertainty will be plotted on cost effectiveness planes, and the probability of the intervention being cost effective will be calculated across different value levels a decision maker would be willing to pay and plotted on cost effectiveness acceptability curves.^51^

### Mediation and moderation analyses

Week-to-week changes in facets of emotion regulation will be tested as mediators of POET effects on mental health symptoms, using parallel process latent growth curve modeling with a competing mediator (SEQ). Candidate moderators such as baseline emotion regulation, symptoms of depression and anxiety (RCADS-47), clinical global symptom severity (CGI-S), NSSI occurrence, global functioning (C-GAS), age, and parental coping (CCNES-A) will be assessed before randomization and investigated as moderators and predictors of treatment outcome (i.e., improvement in symptom severity). Moderated treatment effects will be examined using CLMM that include interaction terms between POET and the candidate moderators.

### Long-term and distal outcomes data

Additional analyses of primary and secondary outcomes at 1-year and 5-year follow-up will assess whether treatment effects are sustained over the long term, using the same statistical analysis method applied at the primary endpoint. Data on distal outcomes will be obtained from Swedish national registries. This data will vary in type and distribution such as continuous (e.g., grade point average), binary (e.g., eligibility for higher education), count (e.g., number of health care contacts), and time-to-event (e.g., suicide), appropriate regression models will be fitted accordingly.

# Methods: Monitoring

## Trial monitoring

We will use internal monitoring as part of the quality control in this study. The monitoring process ensures that the rights, safety, and well-being of research participants are protected and that the collected data are accurate. This is done by reviewing compliance with the study protocol, applicable laws and regulations, and by ensuring that study data are recorded in a correct and traceable manner.

## Quality Control and Quality Assurance

Although this trial is not a pharmaceutical trial, it will adhere to the main Good Clinical Practice principles. The trial will be performed in compliance with this clinical trial protocol and current national regulations governing this clinical trial. This is to ensure the safety and integrity of the trial subjects as well as the quality of the data collected. In the event of harm resulting from participation in the study, participants are covered by applicable national patient injury insurance, in accordance with local regulations.

# Ethics

The study has obtained ethical approval from the Swedish Ethical Review Authority (approval no. 2023-03652-0) and was pre-registered before recruitment commenced at ClinicalTrials.gov (ID: NCT06067165). Eventual protocol amendments will be reported to the Swedish Ethical Review Authority and registered at ClinicalTrials.gov. All participants will provide informed consent. Participants aged 15 years or older and parents will provide written consent. Younger participants will provide verbal consent with parental written consent.

## Benefit-risk evaluation

No immediate negative effects are expected as a possible consequence of the treatment programs or assessment procedures. Extensive follow-up period may be perceived as burdensome, but it is considered a necessary part of the evaluation of the treatments. The risk of discomfort during the assessment process is judged to be less serious than the risk of including unsuitable participants in the treatments. The risks and burdens identified in relation to study participation are few and can largely be mitigated through clear information and continuous monitoring of participants’ well-being. On the other hand, the potential benefits of the project are substantial. This study has the potential to contribute valuable knowledge on how scalable, structured, and accessible treatments can be implemented within routine healthcare. The study also offers direct benefits to participants by providing prompt access to free psychological treatment, including weekly therapist support, with the potential for symptom reduction and decreased suffering.

## Measures to minimize risks for participants

Acute problems (e.g., deterioration of mental health problems) may occur. To manage this, weekly contact with a therapist is included as part of the treatment (regardless of treatment condition), and families also have the opportunity to reach out on their own initiative. Families experiencing acute problems will be actively supported in obtaining appropriate care. Therapists will also review participants’ self-assessments weekly to identify any deterioration in well-being that has not been communicated during treatment. Participants who continue to need psychological treatment after the 6-week intervention will be offered referral to appropriate services within regular care in their region.

Being excluded from the study may be perceived as negative. Therefore, clear information about the inclusion and exclusion criteria is available on the project’s website. In cases of exclusion, participants will receive personal feedback and, when appropriate, referral to adequate care.

Clear, age-appropriate information about the project is provided to all potential participants. Both adolescents and parents/guardians are informed that they may withdraw from the study at any time, and they receive information about confidentiality and data handling.

# Handling of Adverse Events

## Definition: Adverse Event (AE)

Adverse Event (AE): Any untoward medical occurrence in a subject to whom a medicinal product is administered and which does not necessarily have a causal relationship with this treatment.

## Definition: Serious Adverse Event (SAE)

Serious Adverse Event (SAE): Any untoward medical occurrence that at any dose requires inpatient hospitalization or prolongation of existing hospitalization, results in persistent or significant disability or incapacity, is life-threatening, or results in death.

## Assessment of Adverse Events (AE)

Participants are asked to report adverse events during treatment period and rate the discomfort of the eventual adverse events caused by their participation in the treatment. These questions will be administered to adolescents immediately after treatment.

During treatment, AE and SAR are registered by a clinician. All AE that occurs during the trial and which are observed by a clinician or reported by the subject will be registered regardless of whether they are assessed as related to the treatment of not. Assessment of causal relationship, severity (mild, moderate, severe), and whether the AE is considered to be an SAE will be made by the project investigator. For each AE/SAE, a description of the event is recorded, start date for the AE/SAE, planned actions, actions taken, causal relationship, and whether it is an AE or SAE. Serious Adverse Events (SAE) are reported to the sponsor within 24 hours of the investigator being informed of the SAE.

### Assessment of causal relationship

The principal investigator is responsible for determining whether there is a causal relationship between the AE/SAE and the treatment.

All AE can be categorized as either likely related, possibly related, or not related.

**Not related:**A clinical event that is reasonably considered not to be related to the intervention. The event is unlikely to be associated with the intervention and can be explained by an underlying medical condition or concomitant medication.

**Possibly related:**A clinical event that occurs within a reasonable time after the intervention has been administered. The event could be explained by the intervention, but there is insufficient information to determine the relationship. The event could also be explained by an underlying condition or medication.

**Probably related:**A clinical event that occurs within a reasonable time after the intervention has been administered. It is unlikely that the event can be attributed to an underlying condition or medication and is most likely caused by the intervention.

### Assessment of expected AE

Based on previous research adverse events are defined a priori as expected (see complete list below). ^45,52^ All other adverse events are defined as unexpected.

Expected Adverse events:

· Increased symptoms of psychological distress (e.g., low mood/anxiety)

· Thoughts about death or suicide

· Increased stress due to the workload of the treatment

· Worsening of well-being during the treatment period

· Increased fatigue

· More or less sleep

· Headache

· Feeling that the treatment is not helping

· Feeling doubtful about the treatment

· Decreased motivation for treatment

· Feeling of not understanding the treatment

· Feelings of shame or guilt when the treatment is not followed as planned

### Assessment of intensity

Each adverse event shall be classified as mild, moderate or severe.

**Mild:** The adverse event is relatively tolerable and transient in its nature but does not affect the subject’s normal life.

**Moderate**: The adverse event causes deterioration of function but does not affect health. The event can be sufficiently unpleasant and interferes with normal activities but does not completely obstruct them.

**Severe**: The adverse event causes deterioration of function or work ability or poses a health risk to the subject.

## **Table 3.** Content of the adolescent program in POET Treatment

| **Module** | **Name and theme of module** | **Content** |
| --- | --- | --- |
| Module 1 | Understanding how emotions work | - Psychoeducation about emotions, functionality of emotions, and emotion regulation and their relationship to mental health problems. - Introduction to fictive example characters. - Introduction to the Process model of emotion regulation. - Homework: Register emotions and identify vulnerability/resilience factors. |
| Video-link session: | Follow-up session | - Goal formulation (specific, measurable, assignable, realistic, and time-related goals). - Address any motivational problems. - Addressing adolescent´s vulnerability/resilience factors. |
| Module 2 | Feeling better by changing or modifying the situation | - Repetition module 1 and review homework. - Addressing unhelpful avoidance. - Information on how to stay in or seek out situations that are usually avoided and how to seek out positive situations. - Homework: Reduce vulnerability factors, stay in situations that usually are avoided or seek out positive situation. |
| Module 3 | Feeling better by focusing on your emotions | - Repetition module 2 and review homework. - Psychoeducation about the components of emotions and addressing emotional awareness. - Homework: Practicing identification of emotions. |
| Module 4 | Feeling better by letting go of or changing your thoughts | - Repetition module 3 and review homework. - Psychoeducation about thoughts, addressing common negative beliefs about emotions and emotion regulation. - Psychoeducation about mindful observations, acceptance of thoughts, and helpful appraisal styles. - Homework: Practicing mindful observation, acceptance of thoughts, and helpful appraisal styles. |
| Module 5 | Feeling better by changing your behaviors | - Repetition module 4 and review homework. - Psychoeducation about behaviors and impulses, addressing impulsivity. - Engage in alternative behaviors in situations when one typically acts impulsively. - Homework: Engage in alternative behaviors instead of engaging in impulsivity. |
| Module 6 | Summary | - Repetition module 4 and review homework. - Summary and maintenance plan. |

## **Table 4.** Content of the parent course in POET Treatment

| **Module** | **Name and theme of module** | **Content** |
| --- | --- | --- |
| Module 1 | Emotions and emotion regulation | - Introduction to emotion regulation and the role of parents during the treatment period. - Introduction to fictive parent characters. - Homework: Supporting the adolescent handling vulnerability/resilience factors. |
| Video-link session: | Follow-up session | - Goal formulation (specific, measurable, assignable, realistic, and time-related goals). - Address any motivational problems. - Addressing adolescent´s vulnerability/resilience factors. |
| Module 2 | Supporting adolescents in developing adaptive skills concerning different situations | - Repetition module 1 and review homework. - Addressing unhelpful avoidance. - Introduction to shared parent-adolescent time. - Introduction to strategies for parents to support their adolescents in facing avoided situations and seeking positive experiences. - Homework: Engage in shared parent-adolescent time and supporting adolescents to stay in situations that are avoided or seek out positive situations. |
| Module 3 | Validation | - Repetition module 2 and review homework. - Introduction to the concept of validation. - Homework: Engage in shared parent-adolescent time and validation. |
| Module 4 | Supporting adolescents in developing adaptive skills to manage their thoughts | - Repetition module 3 and review homework. - Introduction to common pitfalls when practicing validation. - Introduction to how parents can support their adolescent in changing their attitude toward their thoughts. - Homework: Engage in shared parent-adolescent time, validation, and supporting their adolescent with acceptance of thoughts and helpful appraisal styles. |
| Module 5 | Supporting adolescents in developing adaptive skills to manage impulsivity and manage conflicts | - Introduction to conflict management and collaborative problem-solving. - Homework: Practice collaborative problem-solving. |
| Module 6 |  | - Summary and maintenance plan |

## **Table 5.** Content of the adolescent program in Supportive Treatment

| **Module** | **Name and theme of module** | **Content** |
| --- | --- | --- |
| Module 1 | Receiving support | - Introduction to mental health. - Reflection on the type and level of support the adolescent needs from oneself and others. - Introduction to fictive characters. - Homework: Reflect on their well-being and mental health problems. |
| Video-link session: | Follow-up session | - Address any motivational problems with adolescent. - Addressing direction for the treatment by reflecting on the “What does improved well-being mean to you?”. |
| Module 2 | Self-esteem, self-confidence, and identity | - Repetition module 1 and review homework. - Introduction to self-esteem, self-confidence, and identity. - Psychoeducation about common mental health problems. - Homework: Reflect on their self-esteem, and self-confidence during the week and how it can be strengthened. |
| Module 3 | Friendships | - Repetition module 2 and review homework. - Psychoeducation about the reasons (genetics, environment) for having common mental health problems. - Introduction to and reflection on friendship and how it can affect one’s mental health. - Homework: Reflect on what defines a good friendship, how they can handle bad relationships and how to find new friends. |
| Module 4 | Family | - Repetition module 3 and review homework. - Psychoeducation about how mental health problems are manifested physically (e.g., headaches, digestive problems, and fatigue). - Introduction to and reflection on family problems and how they might affect one´s mental health. - Homework: Reflect on how the family affects one´s mental health and how to make the best of one´s family situation. |
| Module 5 | School | - Repetition module 4 and review homework. Psychoeducation on reasons for developing mental health issues. - Introduction to and reflection on school and how school might affect one´s mental health. - Homework: Reflect on what can be done in school to positively impact their mental health and how to manage school stress. |
| Module 6 | Summary | - Repetition module 5 and review homework. - Summary and maintenance plan. |

## **Table 6.** Content of the parent course in Supportive Treatment

| **Module** | **Name and theme of module** | **Content** |
| --- | --- | --- |
| Module 1 | Supporting adolescents in reflecting on receiving support | - Introduction to mental health problems in youth the role of parents in treatment. - Reflection on the types of mental health problems affecting their adolescent. - Introduction to fictive parent characters - Homework: Reflect on what could be done to improve the well-being of their adolescent. |
| Video-link session: | Follow-up session | - Address any motivational problems with - adolescent and parent. |
| Module 2 | Supporting adolescents in reflecting on self-esteem, self-confidence, and identity | - Repetition module 1 and review homework. - Introduction to and reflecting on being a parent to an adolescent. - Homework: Reflect on the challenges they experience in parenting and on how to strengthen their adolescent’s self-esteem. |
| Module 3 | Supporting adolescents in reflecting on friendship | - Repetition module 2 and review homework. - Introduction to and reflecting on their adolescent safety net. - Introduction to and reflection on their adolescent´s internet use. - Homework: Reflect on how they can support their adolescent's friendships. Reflect on concerns about their adolescent’s internet use. |
| Module 4 | Supporting adolescents in reflecting on family relations | - Repetition module 3 and review homework. - Introduction to and reflecting on their adolescent family relations. - Introduction to and reflection on self-care activities. - Homework: Reflect on actions to take to improve family relationships, and self-care activities for themselves. |
| Module 5 | Supporting adolescents in reflecting on school | - Repetition module 4 and review homework. - Introduction to and reflection on how school might affect one´s mental health. - Homework: The parent is encouraged to reflect on their adolescent school situation and self-care activities for themselves. |
| Module 6 | Summary | - Repetition module 5 and review homework. - Summary and maintenance plan. |

# References

1. Roberts RE, Fisher PW, Blake Turner J, Tang M. Estimating the burden of psychiatric disorders in adolescence: the impact of subthreshold disorders. Soc Psychiatry Psychiatr Epidemiol. 2015 Mar 1;50(3):397–406.

2. Lewinsohn PM, Shankman SA, Gau JM, Klein DN. The prevalence and co-morbidity of subthreshold psychiatric conditions. Psychological Medicine. 2004 May;34(4):613–22.

3. Patton GC, Sawyer SM, Santelli JS, Ross DA, Afifi R, Allen NB, et al. Our future: a Lancet commission on adolescent health and wellbeing. The Lancet. 2016 June;387(10036):2423–78.

4. Merikangas KR, He J ping, Burstein M, Swanson SA, Avenevoli S, Cui L, et al. Lifetime Prevalence of Mental Disorders in US Adolescents: Results from the National Comorbidity Study-Adolescent Supplement (NCS-A). J Am Acad Child Adolesc Psychiatry. 2010 Oct;49(10):980–9.

5. Marchette LK, Weisz JR. Practitioner Review: Empirical evolution of youth psychotherapy toward transdiagnostic approaches. Journal of Child Psychology and Psychiatry. 2017;58(9):970–84.

6. Radez J, Reardon T, Creswell C, Lawrence PJ, Evdoka-Burton G, Waite P. Why do children and adolescents (not) seek and access professional help for their mental health problems? A systematic review of quantitative and qualitative studies. Eur Child Adolesc Psychiatry. 2020/01/21 edn 2021 Feb;30(2):183–211.

7. Swedish Association of Local Authorities and Regions. Första linjen för barns och ungas psykiska hälsa 21/22 [Internet]. Stockholm: Swedish Association of Local Authorities and Regions; 2022 [cited 2025 Dec 8]. Available from: https://www.uppdragpsykiskhalsa.se/wp-content/uploads/2022/06/La%CC%88gesrapport-Fo%CC%88rsta-linjen-2022_final_.pdf

8. Insel T, Cuthbert B, Garvey M, Heinssen R, Pine DS, Quinn K, et al. Research Domain Criteria (RDoC): Toward a New Classification Framework for Research on Mental Disorders. AJP. 2010 July;167(7):748–51.

9. Sheppes G, Suri G, Gross JJ. Emotion Regulation and Psychopathology. Annual Review of Clinical Psychology. 2015;11(1):379–405.

10. Moltrecht B, Deighton J, Patalay P, Edbrooke-Childs J. Effectiveness of current psychological interventions to improve emotion regulation in youth: a meta-analysis. Eur Child Adolesc Psychiatry. 2021 June;30(6):829–48.

11. Sheehan DV, Lecrubier Y, Sheehan KH, Amorim P, Janavs J, Weiller E, et al. The Mini-International Neuropsychiatric Interview (M.I.N.I.): the development and validation of a structured diagnostic psychiatric interview for DSM-IV and ICD-10. J Clin Psychiatry. 1998;59 Suppl 20:22-33;quiz 34-57.

12. Gross JJ. The Emerging Field of Emotion Regulation: An Integrative Review. Review of General Psychology. 1998 Sept 1;2(3):271–99.

13. Gross JJ. Emotion Regulation: Current Status and Future Prospects. Psychological Inquiry. 2015 Jan 2;26(1):1–26.

14. Bjureberg J, Ojala O, Hesser H, Häbel H, Sahlin H, Gratz KL, et al. Effect of Internet-Delivered Emotion Regulation Individual Therapy for Adolescents With Nonsuicidal Self-Injury Disorder: A Randomized Clinical Trial. JAMA Network Open. 2023 July 13;6(7):e2322069.

15. Sjöblom K, Frankenstein K, Klintwall L, Nilbrink J, Zetterqvist M, Hesser H, et al. Online Transdiagnostic Emotion Regulation Treatment for Adolescents With Mental Health Problems: A Randomized Clinical Trial. JAMA Network Open. 2025 June 11;8(6):e2514871.

16. Nordh M, Wahlund T, Jolstedt M, Sahlin H, Bjureberg J, Ahlen J, et al. Therapist-Guided Internet-Delivered Cognitive Behavioral Therapy vs Internet-Delivered Supportive Therapy for Children and Adolescents With Social Anxiety Disorder: A Randomized Clinical Trial. JAMA Psychiatry. 2021 July 1;78(7):705–13.

17. McCauley E, Berk MS, Asarnow JR, Adrian M, Cohen J, Korslund K, et al. Efficacy of Dialectical Behavior Therapy for Adolescents at High Risk for Suicide: A Randomized Clinical Trial. JAMA Psychiatry. 2018 Aug 1;75(8):777–85.

18. Hadjistavropoulos HD, Schneider LH, Klassen K, Dear BF, Titov N. Development and evaluation of a scale assessing therapist fidelity to guidelines for delivering therapist-assisted Internet-delivered cognitive behaviour therapy. Cogn Behav Ther. 2018 Nov;47(6):447–61.

19. Busner J, Targum SD. The Clinical Global Impressions Scale. Psychiatry (Edgmont). 2007 July;4(7):28–37.

20. Shaffer D, Gould MS, Brasic J, Ambrosini P, Fisher P, Bird H, et al. A Children’s Global Assessment Scale (CGAS). Archives of General Psychiatry. 1983 Nov 1;40(11):1228–31.

21. Jassi A, Lenhard F, Krebs G, Gumpert M, Jolstedt M, Andrén P, et al. The Work and Social Adjustment Scale, Youth and Parent Versions: Psychometric Evaluation of a Brief Measure of Functional Impairment in Young People. Child Psychiatry Hum Dev. 2020 June;51(3):453–60.

22. Chorpita BF, Yim L, Moffitt C, Umemoto LA, Francis SE. Assessment of symptoms of DSM-IV anxiety and depression in children: a revised child anxiety and depression scale. Behaviour Research and Therapy. 2000 Aug;38(8):835–55.

23. Lenhard F, Mitsell K, Jolstedt M, Vigerland S, Wahlund T, Nord M, et al. The Internet Intervention Patient Adherence Scale for Guided Internet-Delivered Behavioral Interventions: Development and Psychometric Evaluation. J Med Internet Res. 2019 Oct 1;21(10):e13602.

24. Radez J, Waite P, Chorpita B, Creswell C, Orchard F, Percy R, et al. Using the 11-item Version of the RCADS to Identify Anxiety and Depressive Disorders in Adolescents. Res Child Adolesc Psychopathol. 2021;49(9):1241–57.

25. Olderbak S, Uusberg A, MacCann C, Pollak KM, Gross JJ. The Process Model of Emotion Regulation Questionnaire: Assessing Individual Differences in Strategy Stage and Orientation. Assessment. 2023 Oct;30(7):2090–114.

26. Bjureberg J, Ljótsson B, Tull MT, Hedman E, Sahlin H, Lundh LG, et al. Development and Validation of a Brief Version of the Difficulties in Emotion Regulation Scale: The DERS-16. J Psychopathol Behav Assess. 2016 June;38(2):284–96.

27. Gullone E. Gullone, E. & Taffe, J. (2011, October 24). The Emotion Regulation Questionnaire for Children and Adolescents (ERQ-CA): A psychometric evaluation. Psychological Assessment, Advance online publication. doi: 10.1037/a0025777. Psychological Assessment. 2011 Jan 1;

28. Preece DA, Mehta A, Petrova K, Sikka P, Bjureberg J, Chen W, et al. The Perth Alexithymia Questionnaire-Short Form (PAQ-S): A 6-item measure of alexithymia. Journal of Affective Disorders. 2023 Mar 15;325:493–501.

29. Gratz KL. Measurement of Deliberate Self-Harm: Preliminary Data on the Deliberate Self-Harm Inventory. Journal of Psychopathology and Behavioral Assessment. 2001 Dec 1;23(4):253–63.

30. Ebesutani C, Regan J, Smith A, Reise S, Higa-McMillan C, Chorpita BF. The 10-Item Positive and Negative Affect Schedule for Children, Child and Parent Shortened Versions: Application of Item Response Theory for More Efficient Assessment. J Psychopathol Behav Assess. 2012 June 1;34(2):191–203.

31. Muris P. A Brief Questionnaire for Measuring Self-Efficacy in Youths. Journal of Psychopathology and Behavioral Assessment. 2001 Sept 1;23(3):145–9.

32. Bohus M, Limberger MF, Frank U, Sender I, Gratwohl T, Stieglitz RD. [Development of the Borderline Symptom List]. Psychother Psychosom Med Psychol. 2001 May;51(5):201–11.

33. Becerra R, Preece DA, Gross JJ. Assessing beliefs about emotions: Development and validation of the Emotion Beliefs Questionnaire. Blanch A, editor. PLoS ONE. 2020 Apr 14;15(4):e0231395.

34. Chen G, Stevens K, Rowen D, Ratcliffe J. From KIDSCREEN-10 to CHU9D: creating a unique mapping algorithm for application in economic evaluation. Health Qual Life Outcomes. 2014 Aug 29;12:134.

35. Fabes, R. A., Eisenberg, N., & Bernzweig, J. Coping with Children’s Negative Emotions Scale (CCNES): Description and scoring. Tempe, AZ: Arizona State University; 1990.

36. Kraaij V, Garnefski N. The Behavioral Emotion Regulation Questionnaire: Development, psychometric properties and relationships with emotional problems and the Cognitive Emotion Regulation Questionnaire. Personality and Individual Differences. 2019 Jan;137:56–61.

37. Hakkaart-van Roijen L, Van Straten A, Donker M, Tiemens B. Manual Trimbos/iMTA questionnaire for costs associated with psychiatric illness (TIC-P). Institute for Medical Technology Assessment. 2002 Jan 1;

38. Devilly GJ, Borkovec TD. Psychometric properties of the credibility/expectancy questionnaire. Journal of Behavior Therapy and Experimental Psychiatry. 2000 June 1;31(2):73–86.

39. Attkisson CC, Zwick R. The client satisfaction questionnaire. Psychometric properties and correlations with service utilization and psychotherapy outcome. Eval Program Plann. 1982;5(3):233–7.

40. Hayes AF, Krippendorff K. Answering the Call for a Standard Reliability Measure for Coding Data. Communication Methods and Measures. 2007 Apr;1(1):77–89.

41. Hopewell S, Chan AW, Collins GS, Hróbjartsson A, Moher D, Schulz KF, et al. CONSORT 2025 explanation and elaboration: updated guideline for reporting randomised trials. BMJ. 2025 Apr 14;389:e081124.

42. R: The R Project for Statistical Computing [Internet]. [cited 2024 Aug 2]. Available from: https://www.r-project.org/

43. Feingold A. New Approaches for Estimation of Effect Sizes and their Confidence Intervals for Treatment Effects from Randomized Controlled Trials. Quant Method Psychol. 2019;15(2):96–111.

44. Little, Roderick J. A., Rubin, Donald B. Statistical analysis with missing data. 2nd ed. Hoboken, N.J: Wiley; 2002.

45. Grudin R, Ahlen J, Mataix-Cols D, Lenhard F, Henje E, Månsson C, et al. Therapist-guided and self-guided internet-delivered behavioural activation for adolescents with depression: a randomised feasibility trial. BMJ Open. 2022 Dec 26;12:e066357.

46. Andrén P, Aspvall K, Cruz LF de la, Wiktor P, Romano S, Andersson E, et al. Therapist-guided and parent-guided internet-delivered behaviour therapy for paediatric Tourette’s disorder: a pilot randomised controlled trial with long-term follow-up. BMJ Open. 2019 Feb 1;9(2):e024685.

47. Drummond MF, Sculpher MJ, Claxton K, Stoddart GL, Torrance GW. Methods for the Economic Evaluation of Health Care Programmes. Oxford University Press; 2015. 464 p.

48. Husereau D, Drummond M, Petrou S, Carswell C, Moher D, Greenberg D, et al. Consolidated Health Economic Evaluation Reporting Standards (CHEERS) statement. BMJ. 2013 Mar 25;346:f1049.

49. Stevens K. Valuation of the Child Health Utility 9D Index. PharmacoEconomics. 2012 Aug 1;30(8):729–47.

50. Matthews JN, Altman DG, Campbell MJ, Royston P. Analysis of serial measurements in medical research. BMJ. 1990 Jan 27;300(6719):230–5.

51. Fenwick E, O’Brien BJ, Briggs A. Cost-effectiveness acceptability curves – facts, fallacies and frequently asked questions. Health Economics. 2004;13(5):405–15.

52. Simonsson O, Engberg H, Bjureberg J, Ljótsson B, Stensils J, Sahlin H, et al. Experiences of an Online Treatment for Adolescents With Nonsuicidal Self-injury and Their Caregivers: Qualitative Study. JMIR Form Res. 2021 July 23;5(7):e17910.

##

##

##

##

##

##

## 
