# Supplementary material for: Randomised superiority trial evaluating an online transdiagnostic emotion regulation treatment for adolescents with mental health conditions: study protocol
Source: BMJ Open. 2026 Jul 29;16(7):e116511. doi: 10.1136/bmjopen-2026-116511 (PMC13422934; doi:10.1136/bmjopen-2026-116511)
Supplement: Supplementary file 2 [file bmjopen-2026-116511.reviewer_comments.pdf]

PEER REVIEW HISTORY

BMJ Open publishes all reviews undertaken for accepted manuscripts. Reviewers are asked to complete a checklist review form and are provided with free text boxes to elaborate on their assessment. These free text comments are reproduced below.

ARTICLE DETAILS

Title (Provisional)

A Randomized Superiority Trial Evaluating an Online Transdiagnostic Emotion Regulation Treatment for Adolescents with Mental Health Conditions: Study Protocol

Authors

Sjöblom, Katja; Bellander, Martin; Nilbrink, Jannike; Zetterqvist, Maria; Sampaio, Filipa; Hedman-Lagerlöf, Erik; Hellner, Clara; Gross, James J.; Hesser, Hugo; Bjureberg, Johan

---

VERSION 1 - REVIEW

**Reviewer**	**1**
**Name**	**Mayer, Gwendolyn**
**Affiliation**	**University Hospital Heidelberg, Internal Medicine and Psychosomatics**
**Date**	**17-May-2026**
**COI**	**None**

This protocol describes a single-blind RCT with the aim to prove whether an online emotion regulation intervention for adolescents with mental health problems is superior to an active control treatment. The protocol is well-written and structured. Overall, only few questions remained open to me after reading the paper:

Abstract: The methods are written in future tempus, as expected for a protocol, with the exception of "participants are 388 adolescents" – which leads to the question, why the recruitment aims (power analysis) bring this weird number – could be anything above 385?

Strengths and limitations: so far, I read only about strengths – all studies have limitations…

Is there a specific definition of adolescence that guided the development of the protocol and the choice of the age group 12-17? There are other definitions ranging 10-17 or even 10-24.

The protocol in the supplement provides a much better explanation regarding lacks in the literature regarding the role of emotion regulation and psychopathology – I suggest to add at least one short reflection to the paper as well

Gender differences and sexual orientation both play a significant role in the development of mental disorders in the critical time of puberty, how was this regarded in the concept of the study

Line 100: the mediation and moderation analyses are methods, no aims. The related aims could be: understand the temporal roles of emotion regulation in mental health or understand reasons for interpersonal differences etc.

How is self-harm considered in adverse events monitoring?

Line 171: planned interventions (should be in plural)

The description of the control intervention remains vague, focusses on general requirements about rigor and internal validity – after reading both paragraphs I did not really understand the expectation that POET will be superior to the control intervention on general mental health. Few more sentences on the differences would be helpful

Was parental mental health assessed?

The reimbursement should not be left to the supplement as it explains the low attrition rate that was expected

Are there any anticipated results of this study that can already be reported and discussed, how the study will contribute to the role of emotion regulation in adolescence?

VERSION 1 - AUTHOR RESPONSE

Reviewer 1

RC1: This protocol describes a single-blind RCT with the aim to prove whether an online emotion regulation intervention for adolescents with mental health problems is superior to an active control treatment. The protocol is well-written and structured. Overall, only few questions remained open to me after reading the paper:

AR: Thank you for your encouraging remarks. We are pleased to hear that the protocol was found to be well-written and structured. Below, we address the remaining questions.

RC2: Abstract: The methods are written in future tempus, as expected for a protocol, with the exception of “participants are 388 adolescents” – which leads to the question, why the recruitment aims (power analysis) bring this weird number – could be anything above 385?

AR: We thank the reviewer for giving us the opportunity to clarify this point. The number of 388 participants derives from our simulation-based power analysis, which indicated that 350

participants would provide >80% power to detect an interaction between treatment and time with an odds ratio of 2.2. To account for an estimated 10% attrition rate, the sample size was adjusted to 388. In addition, we have revised the abstract to clarify that we aim to include 388 participants.

Abstract, Page 2, Lines 35-37:

[We aim to include 388 participants (adolescents aged 12 to 17 years with mental health conditions and their parents), recruited through primary care and self-referral.]

RC4: Strengths and limitations: so far, I read only about strengths – all studies have limitations…

AR: We thank the reviewer for this observation. We have revised the 'Strengths and limitations of this study' section to include both strengths and limitations of our methodology. Please see our response to ED3.

RC5: Is there a specific definition of adolescence that guided the development of the protocol and the choice of the age group 12-17? There are other definitions ranging 10-17 or even 10-24.

AR: We acknowledge that definitions of adolescence vary. The selected age range represents a developmental period during which adolescents are generally able to engage in treatment independently, whereas younger children may require a more active and co-therapeutic role of parents, particularly in treatment delivered online.2 Including older adolescents (18–24 years) would likely necessitate a different treatment approach given their different developmental needs and life circumstances. The age range further align with the feasibility trial,3 and reflects a pragmatic decision to target the age group with the highest proportion of visits to primary care in Sweden.4

Methods and analysis, Page 7, Lines 127-130:

[The selected age range of 12–17 years reflects both developmental and pragmatic considerations, excluding younger adolescents who would require more extensive parental involvement and older adolescents who would likely prefer to engage more independently in treatment]

RC6: The protocol in the supplement provides a much better explanation regarding lacks in the literature regarding the role of emotion regulation and psychopathology – I suggest to add at least one short reflection to the paper as well

AR: We have added a brief reflection in the introduction acknowledging that intervention studies are needed to better understand the relationship between psychopathology and emotion regulation.

Introduction, Page 5, Lines 77-81:

[Although both prospective and experimental findings suggest that emotion regulation may play an important role in the emergence and maintenance of psychopathology, the nature of this relationship remains unclear. Intervention studies that experimentally manipulate emotion regulation processes, such as randomized controlled trials targeting emotion regulation, may be particularly valuable in clarifying this relationship.9]

RC7: Gender differences and sexual orientation both play a significant role in the development of mental disorders in the critical time of puberty, how was this regarded in the concept of the study

AR: We thank the reviewer for raising this important point. Gender identity will be assessed in an inclusive manner, offering response options beyond the gender binary, including the alternatives non-binary, other, unsure, and prefer not to answer. Data on gender identity will be collected in the study, and the sample size will allow for explorative subgroup analyses to explore potential differences in treatment response. In addition, we have strived to incorporate a gender-neutral and non-heteronormative perspective in the development of the treatment. For example, one of the fictional characters featured in the interventions has a gender-neutral name and is depicted as having a sexual orientation that reflects diversity beyond heterosexuality.

Methods and analysis, Page 10, Lines 191-193:

[In addition, we have strived to incorporate a gender-neutral and non-heteronormative perspective in the development of the interventions.]

Methods and analysis, Page 12, Lines 240-241:

[Adolescents will report on gender identity, assessed inclusively with response options extending beyond the gender binary.]

RC8: Line 100: the mediation and moderation analyses are methods, no aims. The related aims could be: understand the temporal roles of emotion regulation in mental health or understand reasons for interpersonal differences etc.

AR: We agree that mediation and moderation refer to analytical approaches rather than aims. We have therefore revised the wording to clarify the secondary aims of the study.

Introduction, Page 6, Lines 100-104:

[Additional secondary aims, detailed in the trial protocol in the Supplement, include examining whether improvements in emotion regulation is a potential mechanism of change, identify factors that influence for whom and under what conditions the treatment is most effective, and further assessments of the durability of treatment effects and other distal outcomes.]

RC9: How is self-harm considered in adverse events monitoring?

AR: Thank you for this important question. Self-harm will be captured within our adverse events (AE) monitoring in several ways. First, the self-reported AE measure is intentionally broad, allowing participants to report any negative experiences during the study period, including self-harm. In addition, clinicians are instructed to register and actively follow up on any indications of worsening mental health or negative events during the treatment period.

For patient safety, blind assessors are specifically instructed to alert the study coordinator if self-harming behaviors are present. In addition, self-harming behavior is monitored during follow up assessment points using the Deliberate Self-Harm Inventory, Youth version.5 If new onset or escalating self-harm is identified, the family is contacted promptly by the research team. A key strength of our approach is that adverse events, including self-harm, are assessed using multiple informants, providing a more nuanced and comprehensive picture of the presence and of AEs.

The manuscript has been revised to clarify the procedures for monitoring self-harm.

Methods and analysis, Page 9, Lines 173-177:

[Self-harm will be monitored throughout the study using multiple methods, including clinician registration of negative events, such as self-harm during treatment and an adolescent self-reported adverse events measure administered after treatment. The Deliberate Self-Harm Inventory, Youth version,5 will be administered at follow-up assessments, and blind assessors will alert the study coordinator if new onset or escalating self-harm is identified.]

RC10: Line 171: planned interventions (should be in plural)

AR: We have revised accordingly.

RC11: The description of the control intervention remains vague, focusses on general requirements about rigor and internal validity – after reading both paragraphs I did not really understand the expectation that POET will be superior to the control intervention on general mental health. Few more sentences on the differences would be helpful

AR: Thank you for this helpful comment. We have now revised the manuscript, and we hope this revision make the distinction between the interventions clearer and to better justify our expectation that POET will have stronger effects on mental health conditions. Specifically, we clarify that supportive treatment does not include any of the active components of POET (e.g., explicit training and therapist guidance in emotion regulation strategies, treatment goal setting, or structured practice), but is instead limited to non-specific supportive elements such as self-reflection and general encouragement from the therapist, while being matched in structure and length. We have further clarified that adolescents will receive psychoeducational information on how different life areas may influence mental health, along with general advice on strategies to improve well-being in relation to these areas.

Methods and analysis, Page 11, Lines 215-226:

[Supportive treatment contains no active elements of POET, such as explicit training and therapist support in emotion regulation strategies, treatment goal setting, or structured practice. Rather, it is limited to non-specific element such as self-reflection and general supportive encouragement from the therapist, while being matched to POET in both structure and page count....In the supportive treatment, adolescents are presented with information on mental health and are encouraged to reflect on themes related to well-being such as school, family, self-esteem and peer relationships. For each theme, adolescents receive information on how these areas may influence mental health and are provided with general advice on how to improve their well-being.]

RC12: Was parental mental health assessed?

AR: Data on parental mental health will be collected through self-reported psychiatric diagnoses and the Difficulties in Emotion Regulation Scale-16 (DERS-16).6 A section on demographic background data has been added to the methods and analysis, clarifying that parental psychiatric diagnoses will be collected. The DERS-16 is detailed in the trial protocol and summarized in Table 1.

Methods and analysis, Page 12, Lines 239-241:

[Demographic background data will be collected, including demographics such as parental educational level, occupational status, and country of birth, as well as parental psychiatric diagnoses.]

RC13: The reimbursement should not be left to the supplement as it explains the low attrition rate that was expected

AR: We have incorporated information on reimbursement into the methods section.

Method and analysis , Page 8, Lines 151-154:

[Reimbursement

Adolescents will receive a gift card valued at 100 SEK (approximately £9) upon completion of each assessment following the treatment period, with a maximum total reimbursement of 800 SEK (approximately £72). Participants will not receive any reimbursement during the treatment period.]

RC14: Are there any anticipated results of this study that can already be reported and discussed, how the study will contribute to the role of emotion regulation in adolescence?

We thank the reviewer for this suggestion. We have added a discussion section addressing the broader potential implications of our findings, including their potential to advance understanding of emotion regulation in adolescent mental health and to inform policy and clinical practice.

Discussion , Page 17, Lines 366-373:

[Discussion

This trial has the potential to generate high-quality evidence on a brief, online, transdiagnostic, theory-based intervention targeting emotion regulation in adolescents with mental health conditions. By explicitly targeting emotion regulation, the study may contribute to a better understanding of its role in adolescent mental health. This trial may also inform a scalable and cost-effective approach to delivering evidence-based care in primary care settings. The findings are expected to provide valuable insights for

policymakers, health care providers, and researchers regarding the implementation of psychological treatments for a highly prioritized population.]
